# Tecnologías de modificación corporal y personas travestis y trans en Argentina: un estudio cuantitativo sobre desigualdades en el uso y acceso

**DOI:** 10.18294/sc.2025.5206

**Published:** 2025-03-21

**Authors:** Anahí Farji Neer, María Alejandra Dellacasa, Sebastián Ezequiel Sustas, Melina Antoniucci, Cecilia Rustoyburu, Clara Inés Noceti, Natacha Mateo, Alejandra Rosario Roca

**Affiliations:** 1 Doctora en Ciencias Sociales. Investigadora Asistente, Consejo Nacional de Investigaciones Científicas y Técnicas, con sede en Instituto de Estudios Sociales en Contextos de Desigualdades, Universidad Nacional de José C. Paz, Buenos Aires, Argentina. anahi.farji@gmail.com Consejo Nacional de Investigaciones Científicas y Técnicas Consejo Nacional de Investigaciones Científicas y Técnicas Instituto de Estudios Sociales en Contextos de Desigualdades Universidad Nacional de José C. Paz Buenos Aires Argentina anahi.farji@gmail.com; 2 Doctora en Antropología. Investigadora Asistente, Consejo Nacional de Investigaciones Científicas y Técnicas, con sede en Instituto de Geografía, Historia y Ciencias Sociales, Universidad Nacional del Centro de la Provincia de Buenos Aires, Buenos Aires, Argentina. maledellacasa@yahoo.com.ar Consejo Nacional de Investigaciones Científicas y Técnicas Consejo Nacional de Investigaciones Científicas y Técnicas Instituto de Geografía, Historia y Ciencias Sociales Universidad Nacional del Centro de la Provincia de Buenos Aires Buenos Aires Argentina maledellacasa@yahoo.com.ar; 3 Doctor en Ciencias Sociales. Investigador Asistente, Consejo Nacional de Investigaciones Científicas y Técnicas, con sede en Instituto de Estudios para el Desarrollo Productivo y la Innovación, Universidad Nacional de José C. Paz, Buenos Aires, Argentina. s.sustas@gmail.com Consejo Nacional de Investigaciones Científicas y Técnicas Consejo Nacional de Investigaciones Científicas y Técnicas Instituto de Estudios para el Desarrollo Productivo y la Innovación Universidad Nacional de José C. Paz Buenos Aires Argentina s.sustas@gmail.com; 4 Licenciada en Sociología. Becaria Doctoral, Consejo Nacional de Investigaciones Científicas y Técnicas, con sede en Instituto de Investigaciones sobre Sociedades, Territorios y Culturas, Universidad Nacional de Mar del Plata, Mar del Plata, Argentina. melina.antoniucci@gmail.com Consejo Nacional de Investigaciones Científicas y Técnicas Consejo Nacional de Investigaciones Científicas y Técnicas Instituto de Investigaciones sobre Sociedades, Territorios y Culturas Universidad Nacional de Mar del Plata Mar del Plata Argentina melina.antoniucci@gmail.com; 5 Doctora en Ciencias Sociales. Investigadora Adjunta, Consejo Nacional de Investigaciones Científicas y Técnicas, con sede en Instituto de Investigaciones sobre Sociedades, Territorios y Culturas, Universidad Nacional de Mar del Plata, Mar del Plata, Argentina. ceciliarustoyburu@gmail.com Consejo Nacional de Investigaciones Científicas y Técnicas Consejo Nacional de Investigaciones Científicas y Técnicas Instituto de Investigaciones sobre Sociedades, Territorios y Culturas Universidad Nacional de Mar del Plata Mar del Plata Argentina ceciliarustoyburu@gmail.com; 6 Médica, Especialista en Medicina General y Familiar. Centro de Salud y Acción Comunitaria No. 28, Hospital General de Agudos Donación Francisco Santojanni, Buenos Aires, Argentina. clara.noceti@gmail.com Hospital General de Agudos Donación Francisco Santojanni Centro de Salud y Acción Comunitaria No. 28 Hospital General de Agudos Donación Francisco Santojanni Buenos Aires Argentina clara.noceti@gmail.com; 7 Doctora en Comunicación Social. Becaria posdoctoral, Consejo Nacional de Investigaciones Científicas y Técnicas, con sede en Instituto de Investigaciones sobre Sociedades, Territorios y Culturas, Universidad Nacional de Mar del Plata, Mar del Plata, Argentina. mateonatacha@gmail.com Consejo Nacional de Investigaciones Científicas y Técnicas Consejo Nacional de Investigaciones Científicas y Técnicas Instituto de Investigaciones sobre Sociedades, Territorios y Culturas Universidad Nacional de Mar del Plata Mar del Plata Argentina mateonatacha@gmail.com; 8 Doctora en Antropología. Docente investigadora, Instituto de Estudios para el Desarrollo Productivo y la Innovación, Universidad Nacional de José C. Paz, Buenos Aires, Argentina. roca.ale@gmail.com Universidad Nacional de José C. Paz Instituto de Estudios para el Desarrollo Productivo y la Innovación Universidad Nacional de José C. Paz Buenos Aires Argentina roca.ale@gmail.com

**Keywords:** Personas Transgénero, Identidad de Género, Hormonas, Cirugía, Aceites de Silicona, Argentina

## Abstract

La salud colectiva ha aportado perspectivas analíticas de gran riqueza para comprender la determinación social de la salud y cómo las dinámicas de desigualdad, inequidad e iniquidad son productoras de padecimientos, enfermedades, morbilidad y mortalidad. Desde esta mirada, en este trabajo se identifican y analizan modalidades de apropiación y uso de distintas hormonas e intervenciones de modificación corporal de la población travesti y trans en Argentina, con el propósito de visibilizar las situaciones de vulnerabilidad que puedan estar implicadas en estas prácticas, tanto aquellas realizadas fuera del sistema de salud como las que tuvieron acompañamiento profesional en una institución. Se adoptó una estrategia metodológica cuantitativa, con un diseño descriptivo, observacional y de corte transversal. En 2023, se realizó un relevamiento a través de un cuestionario a nivel federal a personas travestis y trans mayores de 16 años (n=1.196). Los datos analizados dan cuenta de procesos de vulnerabilidad que no se extienden de manera homogénea en toda la población y que responden a desigualdades en salud.

## INTRODUCCIÓN

Las experiencias trans, configuradas en la encrucijada entre los modernos discursos medicalizantes y patologizantes y los de los propios sujetos, tienen profundas vinculaciones con los desarrollos de las tecnologías biomédicas de modificación corporal (tales como las cirugías y los implantes), la consolidación de la endocrinología como especialidad médica y el avance de la industria farmacéutica^1^. Desde mediados del siglo XX hasta comienzos del siglo XXI, especialmente en el contexto europeo y estadounidense, la asignación del diagnóstico de “transexualismo” o “trastorno de la identidad de género” por parte del campo biomédico requirió que los sujetos expresaran de manera inequívoca su deseo y voluntad de modificación de la propia corporalidad a fin de “adecuarla” al género deseado. En esos procesos se legitimó un relato que asociaba las vidas de las personas trans con el padecimiento psíquico y físico. Sin embargo, otros estudios han dado cuenta de narrativas en las que prima la experimentación, el placer o la estética^2^^,^^3^^,^^4^^,^^5^ y han visibilizado que esas exigencias condicionaban a las personas a tener que replicar un guion preestablecido para recibir la autorización para acceder a cirugías y terapias hormonales^6^^,^^7^^,^^8^. Otros abordajes también han dado cuenta que, para las personas trans que desean acceder a ellas, las cirugías y las terapias hormonales constituyen la vía más adecuada para evitar las autolesiones y el sufrimiento, e implican una mejora en su calidad de vida^9^^,^^10^.

La crítica a la patologización de las experiencias trans es uno de los ejes de los *transgender studies* o estudios trans^11^. Este campo tiene su origen en EEUU, en la década de 1990. Sus textos fundacionales elaboraron profundas críticas a los protocolos impuestos por el campo médico para acceder a procedimientos de modificación corporal^8^^,^^12^. Al interior de este campo se forjó el concepto de *privilegio cisexual*^13^ a fin de dar cuenta de un régimen específico que oprime a las personas trans. La histórica desautorización de las voces de las personas trans para hablar de sus cuerpos y sus vidas, constitutiva del cisexismo, ocupa un lugar central en los debates de este campo de estudios^14^.

Desde la década de 2000, en el campo activista, mediante distintas iniciativas y manifestaciones públicas coordinadas en diversos lugares del mundo, comenzó a promoverse que las experiencias trans dejaran de ser patologizadas y entendidas como trastornos, y que el acceso a terapias e intervenciones de modificación corporal no requiriera largos y vejatorios procesos de certificación diagnóstica. A su vez, el activismo reclamaba que dejase de imponerse un único modelo corporal, a fin de que las personas pudieran elegir libremente qué intervenciones realizar, si es que querían realizarlas, y en qué momento de sus trayectorias vitales^15^^,^^16^^,^^17^^,^^18^. Desde estos posicionamientos se abre un debate entre quienes esgrimen el acceso a estas prácticas como una cuestión de salud, y quienes las interpretan como meramente estéticas^19^. Otros posicionamientos pusieron el foco en la autonomía y la agencia de las personas trans, considerando las cirugías como intervenciones que se inscriben en una negociación y en un proceso de democratización del acceso a las tecnologías, trascendiendo la normalización de los cuerpos^20^. En otros trabajos también se ha focalizado en las dimensiones subjetivas de las intervenciones quirúrgicas, recuperando los sentidos y el “renacer” que implican las operaciones para las personas trans^21^ y los aspectos psicológicos y contextuales que influencian la toma de decisiones en torno a las intervenciones corporales de esta población^22^.

### Antecedentes sobre tecnologías de modificación corporal en población travesti y trans en Argentina y América Latina

Las investigaciones etnográficas han sido un aporte fundamental para recuperar las estrategias de construcción de “cuerpos travestis” en diferentes regiones de Argentina, Brasil y Colombia^23^^,^^24^^,^^25^^,^^26^^,^^27^^,^^28^. En esa línea de indagaciones, se ha dado cuenta de la pervivencia de prácticas que, por sus efectos no deseados, pueden resultar peligrosas. Aunque el uso de hormonas sin *supervisión médica* puede acarrear importantes riesgos para la salud^29^^,^^30^^,^^31^^,^^32^^,^^33^, sigue siendo una práctica usual entre las feminidades trans. Así también lo es el uso de inyecciones de siliconas líquidas y/o aceites industriales para modelar los cuerpos, realizado por fuera del sistema de salud formal. Numerosos trabajos han profundizado en los significados culturales, en los motivos por los cuales las personas acceden a estas tecnologías y en los efectos negativos para su salud^27^^,^^34^^,^^35^^,^^36^ y han mostrado cómo se imbrican con procesos de exclusión social. En este sentido, algunos relevamientos cuantitativos han hallado una alta prevalencia de estas prácticas en la población travesti y trans femenina^37^ y las han correlacionado con datos sociodemográficos, laborales y del nivel educativo^38^^,^^39^.

En Argentina, si bien no se habían llevado a cabo estudios cuantitativos de carácter federal con una muestra de tal magnitud, investigaciones previas realizadas por organizaciones travestis y trans y agencias provinciales han aportado indicios de la situación. El relevamiento coordinado por Lohana Berkins en 2007^40^ refiere que el 86% de las feminidades trans encuestadas se habían inyectado siliconas líquidas y el 90% de ellas había realizado el procedimiento en un domicilio particular. También advierte que el 70% había iniciado un proceso de hormonización, pero solo el 20% había adquirido los fármacos a través del sistema de salud. En 2017, una encuesta del Ministerio Público de la Defensa de la Ciudad Autónoma de Buenos Aires^41^ identificó también el uso de siliconas líquidas sin *supervisión médica*, pero notó que el 25% de las travestis y mujeres trans que habían realizado tratamientos hormonales accedía a las hormonas a través del sistema de salud y un 15% lo realizaba en el subsistema público. Además, notaron diferencias respecto de la identidad de género. Entre las masculinidades trans, el 61% accedió a través del hospital público y solo el 4% se autoadministraba hormonas. En el mismo grupo poblacional, el 88% dijo haber modificado su cuerpo y el 97% recurrió a la hormonización. El 7% se había realizado cirugías genitales y cerca de la mitad se había realizado mastectomías.

En Argentina, los debates antes mencionados impulsaron la aprobación, en 2012, de la Ley 26743 de Identidad de Género cuyo contenido, elaborado por el activismo travesti y trans local, establece que no se requiere ningún tipo de autorización médica, judicial o administrativa ni intervenciones quirúrgicas previas para solicitar el cambio registral de nombre y sexo en los documentos identificatorios. A su vez, la normativa establece que las terapias o intervenciones que tengan como objeto la expresión de la identidad de género, posean cobertura por parte de las instituciones de salud de los tres subsistemas: público, privado y obras sociales. La Ley de Identidad de Género e iniciativas previas como los “Consultorios Amigables para la Diversidad Sexual”, consolidados en 2010^42^^,^^43^^,^^44^^,^^45^, tenían como objetivo incidir desde las políticas públicas en la trama informal y riesgosa en la que se producen esos procesos de modificación corporal.

### Desigualdades en salud, riesgo, vulnerabilidad y autoatención

Desde el campo de la salud colectiva se entiende a los procesos de salud-enfermedad-atención en el marco de relaciones de hegemonía/subalternidad y se considera a los sujetos como agentes activos en la definición de prácticas y saberes en salud^46^^,^^47^^,^^48^. Retomando las epistemologías del sur global, la salud colectiva ha aportado perspectivas analíticas de gran riqueza para comprender la determinación social de la salud en relación con las desigualdades económicas y sociales. En este sentido, la caracterización de las nociones de diferencia, distinción, desigualdad, inequidad e iniquidad resulta central^49^. Desde esta mirada, es posible articular dos perspectivas: por un lado, aquella que reconoce las diferencias *naturales* o *genéticas* que son resultado de la interacción entre dinámicas sociales y biológicas y, por otro, la que reconoce que los procesos sociales de dominación capitalista se encuentran en el origen de las desigualdades materiales. Las desigualdades -en un marco estructural de dominación- constituyen inequidades porque son disparidades evitables e injustas. Pero aquellas que, además, son moralmente inaceptables constituyen iniquidades. Al referir específicamente a la salud, esta perspectiva señala que las dinámicas de desigualdad, inequidad e iniquidad son productoras de padecimientos, enfermedades, morbilidad y mortalidad. A este esquema se incorpora la dimensión simbólico-cultural, dado que los sujetos y grupos afirman sus identidades para distinguirse de otros por medio de gustos, rituales, patrones de conducta y modos de vida específicos que tienen impacto en su salud^50^^,^^51^.

Los factores que determinan las desigualdades en salud están conformados, entonces, por la disponibilidad de recursos materiales, el modo de vida -en tanto instancia que articula estilo de vida y condiciones de vida-, y las posibilidades de acceder a los sistemas de atención en salud. Resulta ineludible, desde esta mirada, atender al rol que ocupa el Estado en la producción o transformación de las desigualdades sociales que impactan en la salud. Las políticas públicas contribuyen a la definición y los contenidos de la ciudadanía, en tanto vehiculizan mecanismos de inclusión/exclusión de determinadas personas dentro de la comunidad política y de los derechos que les corresponden, entre ellos, el derecho a la atención médica^52^^,^^53^. Prestando atención a estos desarrollos, en el presente trabajo entendemos al género, la edad, el nivel educativo y haber realizado o no el cambio registral posibilitado a partir de la sanción de la Ley 26743 de Identidad de Género, como dimensiones que configuran desigualdades en el uso y acceso a las tecnologías de modificación corporal en marcos tanto formales como informales.

En este trabajo, al relevar y analizar datos que incluyen prácticas de modificación corporal realizadas por fuera de instituciones formales con potenciales peligros para la salud, nos interesa retomar las críticas que se plantean desde la salud colectiva a la noción de riesgo producida por la epidemiología tradicional. Se cuestiona la forma de entender al riesgo como un fenómeno de naturaleza estrictamente biológica e individual con características objetivables y cuantificables, soslayando las condiciones sociales de los sujetos. También se discute que, a través de una mirada pretendidamente neutral, tiende a producir sentidos moralizantes y culpabilizantes^54^^,^^55^. Como señalan Castiel y Álvarez-Dardet^54^ es preciso mantener cierta vigilancia crítica respecto de las intervenciones puestas en práctica frente a los accionares peligrosos para el individuo o para su entorno. Si bien deben realizarse intervenciones en favor de la disminución de ciertos daños, estas deben evitar sentidos morales culpabilizantes y realizarse con guías y procedimientos clínicos validados que los sustenten^54^.

A diferencia del enfoque de riesgo, la salud colectiva incorpora la perspectiva de la vulnerabilidad a fin de observar los aspectos individuales, sociales y programáticos que intervienen tanto en la reproducción de la vulnerabilidad en salud como en las estrategias posibles para afrontarla^54^^,^^56^^,^^57^. La dimensión individual refiere a los modos en los que los sujetos cotidianamente construyen relaciones comunitarias, de amistad, familiares y/o profesionales a través de los cuales co-configuran y disputan significados sociales. La dimensión social pone el foco en las condiciones estructurales, en los discursos y valores que configuran vulnerabilidades individuales. La programática, por último, atiende a las políticas e instituciones de salud predominantemente, pero también a las de educación, cultura o justicia que pueden tanto reproducir como contener, limitar o contrarrestar las condiciones de vulnerabilidad de los sujetos.

La salud colectiva apunta, entonces, a entender a los procesos de salud-enfermedad-atención a través de una mirada interdisciplinaria, como una totalidad que incluye los modos de vida, las prácticas y los significados construidos por los sujetos cuyas condiciones de salud se ven afectadas^58^. Desde el enfoque adoptado en este trabajo consideramos las prácticas de modificación corporal como constitutivas del modo de vida de la población travesti y trans, que se configuran a partir de significados sobre el cuerpo y la identidad construidos colectivamente y que tienen distintos efectos en las dinámicas de salud-enfermedad-atención.

Con relación a las prácticas e intervenciones que en ocasiones se desarrollan por fuera de contextos asistenciales formales, resulta de interés retomar también aquello que Eduardo Menéndez caracteriza como procesos de autoatención^59^: la articulación de distintos saberes y formas de atención llevadas a cabo por los sujetos y grupos sociales como agentes activos en la construcción de su salud. Estas prácticas deben ser tenidas en cuenta por los servicios de salud formales para elaborar estrategias más eficaces de atención^59^. Ello conlleva un desafío, teniendo en cuenta que, debido al sesgo heredado del paradigma hegemónico del sector salud, las demandas en salud que no logran ser objetivadas dentro de procesos patológicos, presentan dificultades para ser reconocidas como necesidades de salud^60^. A este sesgo se suman los sentidos cisexistas que rigen culturalmente y que no reconocen como legítimos los deseos corporales y proyectos de vida de las personas travestis y trans. En el campo de la salud, y en otros campos profesionales, estos sentidos se encuentran presentes en los contenidos que se transmiten de manera constante y acrítica en la formación de sus profesionales^61^. En este sentido, encontramos más adecuada la noción de “acompañamiento” en lugar de la de “supervisión médica”, ya que esta modalidad contempla las trayectorias particulares de las personas, su agencia y el reconocimiento de los saberes producidos colectivamente por parte de esta población.

Atendiendo a estos desarrollos y discusiones, en este artículo se identifican y analizan modalidades de apropiación y uso de distintas hormonas e intervenciones de modificación corporal de la población travesti y trans en Argentina en 2023, con el propósito de visibilizar las situaciones de vulnerabilidad que puedan estar implicadas en estas prácticas, tanto aquellas realizadas fuera del sistema de salud como las que tuvieron acompañamiento profesional en una institución.

Asimismo, se ha contemplado indagar sobre diversos procedimientos de modificación corporal porque, tal como lo han afirmado otros estudios^4^^,^^62^, si bien las cirugías genitales continúan siendo relevantes, han perdido centralidad como marcadoras de género y forman parte de un abanico mayor de intervenciones. De esta manera, la voz, el cabello, el tórax y los senos, los huesos, la piel o el rostro se leen como indicadores de la diferencia sexual posibles de ser modificados. Focalizamos en el uso de hormonas, implantes de siliconas, inyecciones de aceites industriales y/o siliconas líquidas, cirugías genitales, aplicación de bótox, reducción de la nuez de Adán, terapias fonoaudiológicas, entre otras. Nos interesa visibilizar no solo la agencia de las personas travestis y trans en la apropiación y el uso de estas tecnologías, sino también problematizar cómo estas se encuentran mediadas por las desigualdades en salud y cómo se traman en dinámicas específicas de salud-enfermedad-atención.

## MATERIALES Y MÉTODOS

La investigación adoptó una estrategia metodológica cuantitativa, con un diseño descriptivo, observacional y de corte transversal. Se realizó un muestreo no probabilístico e intencional, a partir de la vinculación con referentes y organizaciones de personas travestis y trans de diferentes regiones del país para la construcción de la muestra. Esta estrategia de muestreo suele ser útil para la descripción de fenómenos en poblaciones específicas que presentan dificultades para su abordaje o contacto^63^^,^^64^. Se establecieron cuotas por identidad de género y región del país con base en datos secundarios del Registro Nacional de las Personas (RENAPER)^65^ relativos al cambio registral. Asimismo, se incorporó el criterio etario para obtener variabilidad generacional en los fenómenos indagados. La estrategia para el establecimiento del tamaño muestral combinó maximizar los contactos obtenidos vía “bola de nieve” a partir de vínculos territoriales con organizaciones de personas travestis y trans, servicios de salud focalizados en la atención de personas LGBT, e instituciones de referencia de la población objetivo. El total de personas que completaron el cuestionario fue de 1.196 personas.

El instrumento de relevamiento de datos incluyó campos relativos al acoso policial, diagnóstico y tratamiento de enfermedades crónicas no transmisibles y transmisibles, procesos de hormonización e intervenciones orientadas a la modificación corporal, modalidades de uso, calidad de atención y acceso al sistema de salud por parte de las personas travestis y trans residentes de Argentina. Finalmente, se incluyó un apartado sociodemográfico. 

Para su elaboración, se contemplaron las siguientes instancias: revisión y adaptación de ítems de instrumentos de estudios afines^66^^,^^67^^,^^68^, adaptación de instrumentos previamente validados para la medición del acceso y utilización de los servicios de salud^69^^,^^70^^,^^71^, validación aparente y de contenidos realizada a partir de siete reuniones del equipo de investigación llevadas a cabo, entre julio y septiembre de 2023, junto a integrantes de organizaciones de la sociedad civil de personas travestis y trans, y dos con profesionales de la salud con experiencia en atención a la población travesti y trans de todas las regiones del país incluidas en el estudio. Finalmente, se realizó una prueba piloto del instrumento con personas travestis y trans de las organizaciones participantes. Luego de este proceso, se llegó a una versión definitiva que fue aplicada en el trabajo de campo. Estas instancias permitieron garantizar la legitimidad del instrumento, del proceso de toma de información, y su posterior análisis, contemplando ajustes de frases, secuencias y ordenamiento temático, incorporación de ítems relevantes para la población objetivo -como así también del personal de salud que suele atenderla-, y la correspondencia conceptual y operativa del cuestionario^72^. Para la aplicación del cuestionario en el territorio, se convocó a organizaciones travestis y trans y de trabajo sexual de las distintas regiones del país con el fin de garantizar su participación en todo el proceso de investigación.

El cuestionario se programó en el software en línea Survey Monkey, que permitió la generación de filtros y pases entre preguntas. También posibilitó garantizar el anonimato y la confidencialidad de los datos de las personas que respondieron. Constó de 87 preguntas, incluyendo una de consentimiento informado. El cuestionario empleó, casi en su totalidad, preguntas cerradas, de respuesta simple, múltiple y numéricas. 

El acceso al campo estuvo garantizado por el proceso previo de participación y colaboración con las organizaciones referidas. La programación del instrumento en el software en línea permitió la aplicación del cuestionario de forma individual y cara a cara en dispositivos móviles personales. La organización del trabajo de campo implicó diferentes roles: las personas integrantes del equipo de investigación del proyecto se encargaron del vínculo con organizaciones y seguimiento del campo y, de acuerdo al caudal de casos asignados, se establecieron coordinaciones de campo y personas a cargo de la toma de los cuestionarios en cada organización. Se realizaron capacitaciones para la implementación del instrumento. A cada persona entrevistadora se le brindó gigabytes para navegar en Internet con su celular, el cual fue utilizado para la toma de los cuestionarios.

Para el análisis de los datos obtenidos en el relevamiento, se aplicó un análisis uni y bivariado. Para el análisis bivariado tomamos, como variables independientes, la identidad de género, la edad agrupada, el máximo nivel educativo alcanzado y la realización o no del cambio registral de sexo y nombre en su documento nacional de identidad (DNI). En relación con las variables independientes, el cuestionario incluyó la pregunta por la identidad autopercibida, que tenía las siguientes opciones de respuesta: mujer trans, feminidades trans, travesti, varón trans, masculinidades trans, transgénero, transexual, no binaria, género fluido, otra identidad. A fin de generar una variable de cruce consistente que diera cuenta de la identidad y de las trayectorias de modificación corporal se realizó un reagrupamiento en tres categorías: feminidades trans, masculinidades trans y otras identidades no cis. Esta decisión metodológica también permite establecer comparaciones con estudios previos^41^^,^^66^. La categoría feminidades trans incluyó los casos correspondientes a su homónima, junto con mujer trans, travesti y casos asignados de la categoría transexual; masculinidades trans incluyó los casos correspondientes a su homónima junto con varón trans y casos de la categoría transexual; otras identidades no cis incluyó transgénero, no binaria, género fluido y otra identidad. El máximo nivel educativo alcanzado lo incorporamos como una variable proximal de la estructuración social. Reagrupamos la variable en tres categorías: quienes tienen hasta primario completo, quienes cursaron secundaria o la terminaron, y quienes accedieron al nivel superior. Asimismo, consideramos el cambio registral como una variable proximal a los soportes de tipo programáticos e institucionales.

Por su parte, las variables dependientes empleadas fueron: haber realizado alguna vez en la vida una terapia de reemplazo hormonal (TRH), la presencia de acompañamiento médico en la primera TRH, quién brindó orientación en caso de no existir acompañamiento médico, la presencia y los tipos de efectos no deseados producto de esa primera TRH, y si las personas encuestadas habían sido informadas de los potenciales efectos no deseados. Otras variables dependientes fueron: haber realizado algún tipo de intervención corporal, los tipos de intervenciones realizadas, la realización de algún tipo de inyección de silicona líquida y/o aceite industrial, si generó algún efecto no deseado y cuáles, la necesidad o intención de realizarse una extracción de la silicona y/o aceites, la intención de realizarse alguna otra intervención corporal y cuáles, y las razones de no haberla realizado aún. Para el procesamiento y análisis de datos se empleó el software SPSS. Con el cálculo de las tablas de contingencia, se realizó una comparación entre las categorías de las variables independientes mediante la prueba de chi cuadrado de Pearson (χ²). Esta prueba no paramétrica es conveniente para el tipo de medición de las variables nominales empleadas, ya que permite valorar si las distribuciones observadas difieren de las esperadas en cada uno de los cruces de las variables dependientes con las variables independientes^63^. El nivel de significación de la prueba estadística fue de valores *p*< 0,05.

Luego de presentar y explicitar la finalidad de la investigación, se detallaba el tratamiento anónimo y confidencial de los datos relevados. Seguidamente, se incluía una pregunta acerca del consentimiento para responder el cuestionario. También estuvo disponible una hoja de información ampliatoria. Si bien no se solicitaron datos personales, se garantizó que no se duplicaran las respuestas a través de la fecha en que se completaba el cuestionario, la edad, el género y lugar de nacimiento. El equipo de investigación no tuvo acceso a información que permita la identificación de las personas participantes. El proyecto fue aprobado el 28 de agosto 2023 por el Comité de Bioética de la Universidad Nacional de Mar del Plata, Buenos Aires, Argentina.

## RESULTADOS

### Descripción de la muestra

El tamaño final de la muestra fue de 1.196 personas (Tabla 1). Cerca de un tercio de ellas residía en el Área Metropolitana de Buenos Aires, un 39,1% en la región centro, y un 8,9% en la región del noroeste. Las regiones Noreste, Cuyo y Patagonia concentraban, cada una, un 6% de las personas que respondieron el cuestionario. Un 59,3% se identificaba como feminidades trans, un 25,4% como masculinidades trans y un 15,3% como otras identidades no cis. La distribución etaria podría relacionarse con las características propias de los procesos de salud, enfermedad y atención de esta población^41^^,^^67^^,^^68^, que se expresó en una muestra joven: el 60,8% tenía menos de 35 años, el 23,7% entre 35 y 44, el 12,0% entre 45 y 54, y solo un 3,4% superaba los 55 años. Respecto del nivel educativo, un 19,2% alcanzó el nivel primario, un 55,5% el secundario y un 25,3% el superior (más allá de haber finalizado o no el nivel). Finalmente, más de dos tercios de las personas encuestadas habían realizado la tramitación de un nuevo documento de identidad.

**Tabla 1:** Caracterización de la muestra según identidad de género, edad agrupada, región de residencia, máximo nivel educativo alcanzado y realización de cambio registral. Argentina, 2023.

Variables	n	%
**Identidad de género**		
Feminidades trans	709	59,3
Masculinidades trans	304	25,4
Otras identidades no cis	183	15,3
**Edad agrupada**		
16-24	292	24,4
25-34	435	36,4
35-44	284	23,7
45-54	144	12,0
55 y +	41	3,4
**Región**		
Centro	468	39,1
AMBA	410	34,3
NOA	107	8,9
Patagonia	73	6,1
NEA	72	6,0
Cuyo	66	5,5
**Máximo nivel educativo**		
Primario	230	19,2
Secundario	664	55,5
Superior	302	25,3
**Cambio registral**		
Sí	837	70,0
No	359	30,0

Fuente: Elaboración propia con base en datos relevados por la investigación original. AMBA= Área Metropolitana de Buenos Aires; NOA= noroeste argentino; NEA= nordeste argentino.

### Procesos de hormonización

Para adentrarnos en las experiencias de uso y formas de acceso a los procesos de hormonización, entre la población que respondió el cuestionario, indagamos si alguna vez en su vida había realizado una terapia de reemplazo hormonal vinculada a su identidad y expresión de género (Tabla 2). Más de la mitad de las personas respondieron afirmativamente (n=670, 56,0%). Si tomamos la identidad de género como variable independiente, este valor asciende al 67,4% entre las masculinidades trans y al 59,5% entre las feminidades trans, mientras que desciende al 23,5% entre las personas con otras identidades no cis (*p*=0,001). Al considerar el máximo nivel educativo alcanzado, observamos que la proporción de personas que realizaron terapias de reemplazo hormonal aumenta a medida que lo hace el nivel educativo: las personas con nivel primario lo hicieron en un 41,3%, las que poseen nivel secundario en un 56,9% y en un 65,2% quienes accedieron al nivel superior (*p*=0,001). Por último, quienes llevaron a cabo el cambio registral de sus documentos de identidad mencionaron afirmativamente haber realizado una terapia de reemplazo hormonal en un 66,2%. De las personas que no realizaron la rectificación registral, solo un 32,3% realizaron la mencionada terapia (*p*=0,001).

**Tabla 2:** Realización de terapia de reemplazo hormonal alguna vez en la vida según identidad de género, edad agrupada, máximo nivel educativo alcanzado y cambio registral. Argentina, 2023.

Variables	Total (n=1.196, 100,0%)	Realizó TRH (n=670, 56,0%)		No realizó TRH (n=526, 44,0%)		Valor de *p*
	n	n	%	n	%	
**Identidad de género**						
Masculinidades trans	304	205	67,4	99	32,6	0,001
Feminidades trans	709	422	59,5	287	40,5	
Otras identidades no cis	183	43	23,5	140	76,5	
**Edad agrupada (en años)**						
16-24	292	166	56,8	126	43,2	0,431
25-34	435	255	58,6	180	41,4	
35-44	284	154	54,2	130	45,8	
45-54	144	76	52,8	68	47,2	
55 y +	41	19	46,3	22	53,7	
**Máximo nivel educativo alcanzado**						
Primario	230	95	41,3	135	58,7	0,001
Secundario	664	378	56,9	286	43,1	
Superior	302	197	65,2	105	34,8	
**Realizó cambio registral**						
No	359	116	32,3	243	67,7	0,001
Sí	837	554	66,2	283	33,8	

Fuente: Elaboración propia con base en datos relevados por la investigación original. TRH= Terapia de reemplazo hormonal. Nota: Valor de *p* correspondiente a la prueba de χ^2^.

Indagamos si esa primera terapia hormonal referida tuvo acompañamiento de un equipo de salud (Tabla 3). Del total de las personas con experiencias de hormonización, el 60,0% (n=402) respondió que lo realizaron por primera vez con acompañamiento médico. Si analizamos la relación entre los grupos de edad y este acompañamiento, observamos que a medida que aumenta la edad de las personas, la proporción de personas que recibieron acompañamiento médico es menor (*p*=0,001). Si se considera la identidad de género se advierten importantes diferencias: un 91,2% de las masculinidades trans realizó esa primera terapia hormonal con el acompañamiento de un o una profesional de la salud mientras que, entre las feminidades trans, fue solo un 43,1% (*p*=0,001). Al analizar el máximo nivel educativo alcanzado, observamos que la proporción de personas con experiencias de hormonización con acompañamiento médico dentro de espacios institucionales de salud es mayor a mayor nivel educativo (*p*=0,001). Finalmente, entre las personas que realizaron el cambio registral, una proporción del 62,1% refirió haber tenido acompañamiento médico durante esa primera experiencia, frente al 50,0% que refirió haber tenido acompañamiento, pero no realizaron la rectificación registral (*p*=0,016).

**Tabla 3:** Realización de primera terapia de reemplazo hormonal con acompañamiento médico en una institución de salud según identidad de género, edad agrupada, máximo nivel educativo alcanzado y cambio registral. Argentina, 2023.

Variables	Total (n=670, 100,0%)	Con acompañamiento médico (n=402, 60,0%)		Sin acompañamiento médico (n=268, 40,0%)		Valor de *p*
	n	n	%	n	%	
**Identidad de género**						
Masculinidades trans	205	187	91,2	18	8,8	0,001
Feminidades trans	422	182	43,1	240	56,9	
Otras identidades no cis	43	33	76,7	10	23,3	
**Edad agrupada (en años)**						
16-24	166	137	82,5	29	17,5	0,001
25-34	255	168	65,9	87	34,1	
35-44	154	67	43,5	87	56,5	
45-54	76	26	34,2	50	65,8	
55 y +	19	4	21,1	15	78,9	
**Máximo nivel educativo alcanzado**						
Primario	95	33	34,7	62	65,3	0,001
Secundario	378	221	58,5	157	41,5	
Superior	197	148	75,1	49	24,9	
**Realizó cambio registral**						
No	116	58	50,0	58	50,0	0,016
Sí	554	344	62,1	210	37,9	

Fuente: Elaboración propia con base en datos relevados por la investigación original. Nota: Valor de *p* correspondiente a la prueba de χ^2^.

Entre quienes realizaron alguna vez un proceso de hormonización, en total el 40,0% (n=268) lo hizo sin acompañamiento médico. Al consultarles de quiénes habían recibido orientación o asesoramiento al momento de iniciarla, la amplia mayoría respondió que fueron amigos y/o amigas (77,6%). El resto se distribuye en porcentajes menores al 10% entre persona conocida, persona compañera de la organización, pareja, “madrina”, familiar, farmacéutico o farmacéutica, Internet o redes sociales. 

Al retomar el grupo de personas que alguna vez realizaron una TRH, el 26,0% (n=174) refirió haber experimentado efectos no deseados en su primera terapia de reemplazo hormonal (Tabla 4). Las diferencias en las proporciones son según las distintas variables independientes incluidas: entre grupos de edad, es más alta la proporción de personas que presentaron efectos no deseados entre las personas de mayor edad (sin ser estadísticamente significativa) y entre quienes se incluyen en la categoría otras identidades no cis (37,2%). También mencionan una mayor proporción de efectos no deseados las personas con menor nivel educativo (34,7%). 

**Tabla 4:** Efectos no deseados en realización de la primera terapia de reemplazo hormonal según identidad de género, edad agrupada, máximo nivel educativo alcanzado y cambio registral. Argentina, 2023.

Variables	Total (n=670, 100,0%)	Presentó efectos no deseados (n=174, 26,0%)		No presentó efectos no deseados (n=447, 66,7%)		No recuerda (n=49, 7,3%)		Valor de *p*
	n	n	%	n	%	n	%	
**Identidad de género**								
Feminidades trans	422	127	30,1	265	62,8	30	7,1	0,001
Masculinidades trans	205	31	15,1	159	77,6	15	7,3	
Otras identidades no cis	43	16	37,2	23	53,5	4	9,3	
**Edad agrupada (en años)**								
16-24	166	36	21,7	118	71,1	12	7,2	0,701
25-34	255	66	25,9	173	67,8	16	6,3	
35-44	154	41	26,6	99	64,3	14	9,1	
45-54	76	25	32,9	46	60,5	5	6,6	
55 y +	19	6	31,6	11	57,9	2	10,5	
**Máximo nivel educativo alcanzado**								
Primario	95	33	34,7	48	50,5	14	14,7	0,001
Secundario	378	91	24,1	269	71,2	18	4,8	
Superior	197	50	25,4	130	66,0	17	8,6	
**Realizó cambio registral**								
Sí	554	137	24,7	378	68,2	39	7,0	0,190
No	116	37	31,9	69	59,5	10	8,6	

Fuente: Elaboración propia con base en datos relevados por la investigación original. Nota: Valor de *p* correspondiente a la prueba de χ^2^.

Al indagar cuáles fueron esos efectos no deseados que afectaban su salud, el 38,5% refirió a cambios de estado de ánimo (malestares que aludieron a la ira, ansiedad, depresión o insomnio). Estas razones se presentaron en mayor proporción entre las feminidades trans y otras identidades no cis. Otros tipos de efectos no deseados señalados fueron los trastornos hepáticos y/o estomacales (19,0%), los cuales se presentaron con mayor intensidad entre las feminidades trans. Le siguieron en orden de mención los problemas cutáneos (12,6%), mayormente referidos entre las masculinidades trans, y variaciones en el peso corporal (12,1%). El resto de los motivos aludidos fueron alteraciones sexuales, dolores sin especificar, cambios en la distribución del vello y el cabello, problemas circulatorios, alteraciones hormonales y cambios en la contextura física.

A las personas que tuvieron efectos no deseados durante su primer proceso de hormonización, les consultamos si habían sido informadas sobre los posibles efectos adversos de las terapias hormonales (Tabla 5). Respondió afirmativamente un 43,7% (n=76). Esta proporción fue mayor entre las masculinidades trans (64,5%), las personas que se identifican con otra identidad no cis (62,5%) y quienes tenían entre 16 y 24 años (61,1%). A medida que aumenta la edad de las personas -y también la distancia temporal con esa primera experiencia- la proporción de personas que recibieron información sobre los efectos no deseados es menor. Se encontró una menor proporción de personas que recibieron información entre las feminidades trans (36,2%) y entre las personas con menor nivel educativo (33,3%).

**Tabla 5:** Información recibida sobre efectos no deseados en la realización de la primera terapia de reemplazo hormonal según identidad de género, edad agrupada, máximo nivel educativo alcanzado y cambio registral. Argentina, 2023.

Variables	Total (n=174, 100,0%)	Recibió información (n=76, 43,7%)		No recibió información (n=88, 50,6%)		No recuerda (n=10, 5,7%)		Valor de *p*
	n	n	%	n	%	n	%	
**Identidad de género**								
Feminidades trans	127	46	36,2	75	59,1	6	4,7	0,007
Masculinidades trans	31	20	64,5	9	29,0	2	6,5	
Otras identidades no cis	16	10	62,5	4	25,0	2	12,5	
**Edad agrupada (en años)**								
16-24	36	22	61,1	10	27,8	4	11,1	0,025
25-34	66	33	50,0	30	45,5	3	4,5	
35-44	41	13	31,7	26	63,4	2	4,9	
45-54	25	7	28,0	17	68,0	1	4,0	
55 y +	6	1	16,7	5	83,3	0	0,0	
**Máximo nivel educativo alcanzado**								
Primario	33	11	33,3	20	60,6	2	6,1	0,673
Secundario	91	44	48,4	42	46,2	5	5,5	
Superior	50	21	42,0	26	52,0	3	6,0	
**Realizó cambio registral**								
No	37	16	43,2	20	54,1	1	2,7	0,646
Sí	137	60	43,8	68	49,6	9	6,6	

Fuente: Elaboración propia con base en datos relevados por la investigación original. Nota: Valor de *p* correspondiente a la prueba de χ^2^.

### Otras intervenciones vinculadas a la identidad y la expresión de género

Del total de personas que completaron el cuestionario, un 46,1% (n=551) refirió haber realizado otro tipo de intervención corporal vinculada a la identidad y la expresión de género (Tabla 6). Más de la mitad de las personas que se identificaban como feminidades trans concretaron este tipo de intervenciones (56,3%), aquellas identificadas con masculinidades trans en un 38,2% y otras identidades no cis en un 19,7% (*p*=0,001). Más del 60% de las personas de entre 35 y 54 años respondieron afirmativamente a esta pregunta. También lo hicieron el 51,2% de las personas de más de 55 años. Por el contrario, solamente un 19,2% de quienes tenían entre 16 y 24 años las habían realizado (*p*=0,001). De la totalidad de las personas que efectuaron el cambio registral, el 55,6% había realizado alguna intervención, frente al 24,0% que no lo hicieron (*p*=0,001). No encontramos diferencias significativas al analizar esta dimensión según el máximo nivel educativo alcanzado.

**Tabla 6:** Realización de otras intervenciones de modificación corporal, según identidad de género, edad agrupada, máximo nivel educativo alcanzado y cambio registral. Argentina, 2023.

Variables	Total (n=1.196, 100,0%)	Realizó OIMC (n=551, 46,1%)		No realizó OIMC (n=645, 53,9%)		Valor de *p*
	n	n	%	n	%	
**Identidad de género**						
Masculinidades trans	304	116	38,2	188	61,8	0,001
Feminidades trans	709	399	56,3	310	43,7	
Otras identidades no cis	183	36	19,7	147	80,3	
**Edad agrupada (en años)**						
16-24	292	56	19,2	236	80,8	0,001
25-34	435	215	49,4	220	50,6	
35-44	284	172	60,6	112	39,4	
45-54	144	87	60,4	57	39,6	
55 y +	41	21	51,2	20	48,8	
**Máximo nivel educativo alcanzado**						
Primario	230	103	44,8	127	55,2	0,341
Secundario	664	318	47,9	346	52,1	
Superior	302	130	43,0	172	57,0	
**Realizó cambio registral**						
No	359	86	24,0	273	76,0	0,001
Sí	837	465	55,6	372	44,4	

Fuente: Elaboración propia con base en datos relevados por la investigación original. OIMC= Otras intervenciones de modificación corporal. Nota: Valor de *p* correspondiente a la prueba de χ^2^.

Entre quienes realizaron alguna intervención, los procedimientos mayormente referidos fueron: implantes mamarios (54,8%), mastectomía con reconstrucción pectoral (22,7%), cirugía de modificación facial (21,8%), implantes en los glúteos (18,0%) y aplicación de bótox (14,0%); en tanto la vaginoplastía fue mencionada solamente por un 4,7% y la faloplastia por un 0,5%. Cerca del 72,9% de las personas que se identificaban como feminidades trans se habían colocado implantes mamarios y más del 92,2% de las masculinidades trans se habían realizado una mastectomía con reconstrucción pectoral.

Preguntamos a las personas si tenían pensado realizarse alguna intervención en el futuro (Tabla 7). El 61,4% (n=734) del total de la muestra respondió afirmativamente. Esta proporción aumenta entre las feminidades trans (64,7%) y levemente entre las masculinidades trans (61,8%), pero es menor entre personas con otras identidades no cis, 47,5% (*p*=0,001). Al analizar por grupos de edad, encontramos también una asociación estadísticamente significativa (*p*=0,005) porque, entre los grupos de edad más jóvenes (16 a 24 años), existe una mayor proporción a realizarse intervenciones, y es menor entre las personas mayores de 45 años. Cuando incorporamos el nivel educativo, encontramos un claro patrón: a mayor nivel educativo aumenta la intención de realizarse algún tipo de intervención (*p*=0,001). Al analizar según el cambio registral observamos mayor predisposición a las intervenciones entre quienes sí lo realizaron (64,0%), y menor entre quienes no (55,2%) (*p*=0,004).

**Tabla 7:** Intención de realizarse otras intervenciones de modificación corporal según identidad de género, edad agrupada, máximo nivel educativo alcanzado y cambio registral. Argentina, 2023.

Variables	Total (n=1.196, 100,0%)	Tiene intención de realizar OIMC (n=734, 61,4%)		No tiene intención de realizar OIMC (n=462, 38,6%)		Valor de *p*
	n	n	%	n	%	
**Identidad de género**						
Feminidades trans	709	459	64,7	250	35,3	0,001
Masculinidades trans	304	188	61,8	116	38,2	
Otras identidades no cis	183	87	47,5	96	52,5	
**Edad agrupada (en años)**						
16-24	292	200	68,5	92	31,5	0,005
25-34	435	272	62,5	163	37,5	
35-44	284	165	58,1	119	41,9	
45-54	144	73	50,7	71	49,3	
55 y +	41	24	58,5	17	41,5	
**Máximo nivel educativo alcanzado**						
Primario	230	117	50,9	113	49,1	0,001
Secundario	664	420	63,3	244	36,7	
Superior	302	197	65,2	105	34,8	
**Realizó cambio registral**						
Sí	837	536	64,0	301	36,0	0,004
No	359	198	55,2	161	44,8	

Fuente: Elaboración propia con base en datos relevados por la investigación original. OIMC= Otras intervenciones de modificación corporal. Nota: Valor de *p* correspondiente a la prueba de χ^2^.

Las intervenciones más mencionadas que deseaban realizar fueron: implantes mamarios (35,1%), cirugía de modificación facial (32,8%), mastectomía con reconstrucción pectoral (22,5%), vaginoplastía (18,4%), implante en glúteo (14,4%), nuez de Adán o reducción tiroideocondroplástica (12,9%), y aplicación de bótox (11,2%). Finalmente, se mencionan las faloplastias (9,8%) -con predominancia de masculinidades trans y un porcentaje menor de otras identidades no cis-, y la terapia fonoaudiológica (9,9%), con similar nivel de mención entre las diferentes identidades de género empleadas como variable independiente. En relación con las identidades de género, la elección por los procedimientos entre las masculinidades trans y las feminidades trans se encuentran condicionadas por las representaciones binarias sobre lo que significa un cuerpo masculino y femenino. Entre las otras identidades no cis, muestran intenciones de acceder a cirugías en el tórax (mastectomías o implantes) y a prácticas de modificación facial.

Entre quienes tenían la intención de realizar una intervención con fines vinculados a la construcción de su corporalidad y expresión de género en el futuro, indagamos los motivos por los cuales aún no la realizaron. Encontramos diferentes dimensiones que dan cuenta de potenciales barreras de acceso, así como de postergación por elección personal. Dentro de los principales motivos, las personas encuestadas señalaron: dificultades económicas para el acceso (39,5%), esperas prolongadas para acceder a la intervención (16,5%), percepción de déficit de profesionales para brindar confianza (10,6%) y falta de capacitación y/o de especialistas (5,6%). Entre los obstáculos de tipo personal identificamos el miedo y/o temor a la intervención (13,5%) y el desconocimiento de los mecanismos institucionales para el acceso a la intervención (12,3%). Otras dificultades que mencionaron refieren a experiencias previas propias y de terceras personas con el sector salud: conocimiento de otras personas que no tuvieron los resultados esperados luego de la intervención (4,1%) y malas experiencias en las instituciones de salud (3,4%).

### Inyecciones con aceites y/o silicona líquida

Además de las intervenciones quirúrgicas indagamos en la aplicación de inyecciones de silicona líquida u otro tipo de inyecciones de aceite para modelar el cuerpo (Tabla 8). Entre quienes realizaron alguna intervención, más de la mitad de las personas respondieron afirmativamente haberlas efectuado (50,6%, n=279). Entre las feminidades trans, una proporción del 67,4% había utilizado aceites y/o silicona líquida, y este valor desciende al 27,8% entre las personas con otra identidad de género no cis;de las masculinidades trans ninguna respondió afirmativamente a esta pregunta (*p*=0,001). Tomando la edad agrupada, se observa que las personas a partir de los 35 años responden afirmativamente en mayor proporción y ese valor es aún mayor entre las personas de 55 años y más (81,0%) (*p*=0,001). Al considerar el máximo nivel educativo alcanzado, observamos que la proporción de aplicación de inyecciones de aceites y/o silicona líquida es mayor en los menores niveles educativos (*p*=0,001). Por último, quienes realizaron el cambio registral de sus documentos de identidad mencionan haber utilizado aceites y/o silicona líquida en un 48,6%, en cambio, de las personas que no realizaron la rectificación registral, un 61,6% recurre a las inyecciones para modelar su cuerpo (*p*=0,026).

**Tabla 8:** Realización de inyección o uso de aceites y/o siliconas líquidas, según identidad de género, edad agrupada, máximo nivel educativo alcanzado y cambio registral. Argentina, 2023.

Variables	Total (n=551, 100,0%)	Realizó IASL (n=279, 50,6%)		No realizó IASL (n=272, 49,4%)		Valor de *p*
	n	n	%	n	%	
**Identidad de género**						
Feminidades trans	399	269	67,4	130	32,6	0,001
Masculinidades trans	116	0	0,0	116	100,0	
Otras identidades no cis	36	10	27,8	26	72,2	
**Edad agrupada (en años)**						
16-24	56	13	23,2	43	76,8	0,001
25-34	215	82	38,1	133	61,9	
35-44	172	110	64,0	62	36,0	
45-54	87	57	65,5	30	34,5	
55 y +	21	17	81,0	4	19,0	
**Máximo nivel educativo alcanzado**						
Primario	103	86	83,5	17	16,5	0,001
Secundario	318	164	51,6	154	48,4	
Superior	130	29	22,3	101	77,7	
**Realizó cambio registral**						
Sí	465	226	48,6	239	51,4	0,026
No	86	53	61,6	33	38,4	

Fuente: Elaboración propia con base en datos relevados por la investigación original. IASL= Inyección o uso de aceites y/o silicona líquida. Nota: Valor de *p* correspondiente a la prueba de χ^2^.

A las personas que respondieron afirmativamente que utilizaron inyecciones de silicona y/o aceites, se les preguntó la edad de esa intervención. La totalidad de las personas encuestadas lo hizo antes de los 24 años. Respecto del ámbito en el que se las habían aplicado, el 85,3% de los casos respondió “en un domicilio particular”. Esta situación muchas veces implica la utilización de sustancias no aptas para uso humano, la ausencia de personal adecuadamente capacitado, y un déficit de las condiciones de higiene y asepsia requeridas que involucran grandes riesgos para la salud.

El 32,3% (n=90) de quienes alguna vez se inyectaron aceites y/o silicona líquida manifestó que tuvo algún efecto no deseado después de la intervención. Este porcentaje asciende entre quienes tenían mayor edad, llegando al 58,8% entre las personas de 55 años y más, y al 43,9% entre quienes tenían de 45 a 54 años. Mientras que ninguna de las personas menores de 24 años refirió padecer dichos efectos, entre las de 25 y 34 años los han percibido el 22,0% y entre las de 35 y 44 años el 33,6% (*p*=0,001). No encontramos diferencias significativas según la identidad de género, el máximo nivel educativo alcanzado y el cambio registral.

El instrumento incluyó una pregunta abierta respecto de cuáles fueron esos efectos no deseados. La respondieron 90 personas y casi un tercio de ellas describió varias afecciones, por esto se identificaron 122 efectos que luego agrupamos en siete categorías que refieren a procesos somáticos similares. El 54,4% refirió padecer dolor, inflamación, molestias y/o hinchazón, generalmente en las piernas; el 34,4% aludió sufrir migración de las sustancias y deformidades; el 14,4% contestó que se le han formado manchas azuladas en la piel y el 10,0% granulomas; el 10,0% fiebre/infecciones y el 4,4% ha asociado las inyecciones de aceites y/o silicona a mareos/náuseas/vómitos. En menor proporción se han mencionado dificultades respiratorias, caderas desparejas, várices y problemas intestinales que hemos incluido en la categoría “otros”.

La edad fue una variable relevante para pensar en los efectos no deseados. El dolor, inflamación, molestias y/o hinchazón fue señalado en mayor proporción por quienes tenían 55 años y más, mientras que, en el resto de los grupos etarios, afectó a cerca de la mitad. La migración de las sustancias y deformidades fueron mencionadas en mayor proporción por quienes tenían entre 35 y 54 años.

Identificamos que, entre quienes realizaron esta práctica, el 75,6% no había realizado controles de salud posteriores. Al analizar por el nivel educativo observamos que esta proporción se acentúa entre quienes alcanzaron solo el primario (79,1%), y desciende significativamente entre quienes alcanzaron el superior (51,7%) (*p*=0,007). No encontramos significancia estadística con el resto de las variables de cruce. Cerca de un 40,1% de las personas que se aplicaron aceites y/o silicona líquida respondieron que tenían la necesidad o intención de realizar la extracción quirúrgica de dichas sustancias.

## DISCUSIÓN Y CONCLUSIONES

Desde una perspectiva inscripta en el campo de la salud colectiva y que retoma enfoques provenientes de los estudios trans, entendemos las terapias y otras prácticas de modificación corporal llevadas adelante por la población travesti y trans como intervenciones que apuntan a sostener sus procesos identitarios. Desde este enfoque, a continuación, recapitulamos los aspectos salientes del análisis presentado, atendiendo a las dinámicas de desigualdad, riesgo/vulnerabilidad y autoatención plausibles de ser identificadas.

Como adelantábamos al comienzo, entendemos el género, la edad, el nivel educativo y haber realizado o no el cambio registral como dimensiones que configuran desigualdades en el uso y acceso a las tecnologías de modificación corporal. Desde el campo de la salud colectiva, las desigualdades configuran inequidades en salud porque implican una exposición diferencial a padecimientos, enfermedades y a fenómenos de morbilidad y mortalidad^49^. Los datos de la población travesti y trans que relevamos en esta investigación nos permiten conocer los modos en los que las diferencias de género, edad, nivel educativo y haber realizado o no el cambio registral configuran diferentes usos de las tecnologías de modificación corporal. Los usos de estas tecnologías se vinculan con los deseos y expectativas subjetivas y colectivas en relación con la apariencia corporal y la expresión de género, pero también con la disponibilidad de dichas tecnologías en el sistema de salud y las posibilidades de acceso por parte de las personas usuarias. En este sentido, las personas con mayor nivel educativo accedieron en mayor medida a las terapias hormonales. Esto da cuenta de la existencia de subgrupos al interior de la población travesti y trans en relación con las posibilidades de acompañamiento de los procesos de modificación corporal hormonal, posiblemente vinculados a la desigual distribución de recursos materiales al interior de esta población.

Los datos también muestran diferenciales modalidades de acceso a los dispositivos de atención. Por ejemplo, las relaciones identificadas entre el cambio registral y el acceso a los dispositivos de atención nos permiten aproximarnos a los vínculos entre los sujetos y las instituciones. Las personas que realizaron el cambio registral presentaron un mayor uso y acceso a intervenciones corporales y contaron con mayor seguimiento y control. Asimismo, las personas que no lo realizaron llevaron a cabo con mayor frecuencia intervenciones por fuera del sistema de salud y efectuaron menos controles. Estos datos nos permiten visibilizar qué derechos aún no se efectivizan para la población travesti y trans que no realizó el cambio registral.

En nuestro país, a partir de la sanción de la Ley 26743, el eje de las políticas públicas se ha ido desplazando de la “patologización” y las estrategias “compensatorias”, hacia un discurso que posiciona a las personas travestis y trans como “sujetos activos de derechos”^73^. Sostenemos que el reconocimiento de la identidad jurídica de una persona que exprese cabalmente su género autopercibido es un derecho fundamental que brinda soporte institucional y favorece una ciudadanía plena, en tanto habilita el ejercicio de otros derechos (atención de la salud, educación, trabajo registrado, ingreso y egreso del país, entre otros). Los datos que se desprenden de esta investigación nos permiten pensar en la relación que existe entre el reconocimiento legal de las identidades travestis y trans que rige desde 2012 en virtud de la Ley de Identidad de Género y la apertura de canales de acceso al sistema formal de salud. Es decir, nos permiten dar cuenta de un proceso en el que la sanción de una normativa abona a una transformación de las desigualdades sociales en salud.

Los datos analizados también dan cuenta de procesos de vulnerabilidad que no se extienden de manera homogénea en toda la población. Identificamos que son las feminidades trans, las personas de mayor edad, las de menor nivel educativo y quienes no realizaron el cambio registral las que presentan menores niveles de acceso al acompañamiento médico y mayor cantidad de prácticas que pueden implicar efectos no deseados.

Desde la perspectiva adoptada en este trabajo, a partir de nuestras variables independientes identificamos dimensiones relativas a los procesos de vulnerabilidad que inciden en las exposiciones a prácticas de modificación corporal con potenciales riesgos o daños a la salud. Estas desigualdades observadas no podrían ser comprendidas cabalmente sin remitir a las inequidades estructurales que las generan. Las trayectorias individuales se insertan en ese escenario signado por vulneraciones acumuladas a lo largo del tiempo, las cuales se corporizan en el acceso diferencial al ejercicio del derecho a la salud integral y a los modos de acceso y uso de tecnologías de modificación corporal. Por ejemplo, identificamos mayor utilización de inyecciones de aceites y/o siliconas entre las personas de más edad. Desde una perspectiva generacional, esto implica procesos de salud-enfermedad-atención atravesados por prácticas riesgosas durante más tiempo en sus vidas, lo que estaría vinculado a una acumulación de vulnerabilidades y vulneraciones.

Si bien con diseños y tamaños muestrales diferentes, los antecedentes existentes a nivel nacional^66^^,^^67^^,^^68^ nos permiten encontrar algunas continuidades en prácticas tales como los procesos de hormonización sin acompañamiento profesional y el uso de inyecciones de aceites y/o silicona líquida entre las feminidades trans y, entre ellas, quienes se encuentran en situación de mayor vulnerabilidad socioeconómica e institucional. En cambio, quienes cuentan con mayores soportes institucionales -tanto por haber realizado su cambio registral o haber alcanzado más altos niveles educativos-, presentan perfiles caracterizados por un mayor contacto con el sistema de salud formal. Esto se manifiesta también en los potenciales daños a la salud producto de las secuelas: son los grupos más vulnerados quienes manifiestan en mayor medida padecer efectos no deseados y/o tener necesidad de atención por las secuelas. En relación con las terapias de reemplazo hormonal, los grupos más vulnerados y las feminidades trans manifestaron haber recibido menor información sobre sus posibles efectos no deseados, lo que da cuenta de un déficit en la calidad de la atención. Asimismo, una lectura en clave generacional permite observar una brecha que podría corresponder a cambios en las políticas públicas y su impacto en la organización de los servicios de atención: las generaciones más jóvenes dan cuenta de mayor información sobre los potenciales efectos negativos de esas terapias comunicados por los equipos de salud.

En relación con las modalidades de autoatención^59^ sostenemos que, en la medida en que los procesos de modificación corporal de la población travesti y trans no sean contemplados como necesidades de salud, se habilitan potenciales obstáculos para su acceso de forma acompañada y en un marco institucional que brinde contención a las personas. En ese sentido, si bien la sanción de la Ley de Identidad de Género en Argentina ha implicado un gran avance en materia de derechos y salud, la falta de espacios de atención con profesionales que se hayan capacitado en salud travesti y trans a lo largo del país, así como la escasa disponibilidad de fármacos hormonales y/o prótesis dificultan la posibilidad de asegurar el acceso tal como lo garantiza la ley. Así, según nuestro análisis, los principales motivos por los que las personas que completaron el cuestionario no concretaron las intervenciones de modificación corporal que deseaban realizar en el sistema de salud fueron: dificultades económicas, esperas prolongadas y falta de profesionales de confianza. Como contracara de esto, a lo largo del tiempo, y sobre todo entre las feminidades trans de mayor edad, entre quienes cuentan con menor nivel educativo y menos realización del cambio registral, se ha configurado un repertorio de prácticas realizadas por fuera del sistema de salud.

Entre las personas travestis y trans habitualmente circula información por canales informales -tanto a través de redes sociales, como “de boca en boca”^61^- respecto de qué hormonas consumir, en qué cantidad y frecuencia para obtener ciertos cambios corporales. Esta modalidad ha llevado a una práctica muy extendida de automedicación. Los datos que relevamos dan cuenta de esto: una parte importante de las personas que realizaron una TRH lo hizo sin acompañamiento médico. Al consultarles quiénes les habían orientado o asesorado al momento de iniciarla, la amplia mayoría respondió que fueron amigos y/o amigas. En un sentido similar, entre esta población circula información y se ha popularizado la práctica de aplicarse inyecciones de diferentes sustancias, tanto aceites como silicona líquida no aptos para uso humano. Tal como mencionamos al comienzo, y como refieren otros trabajos^37^^,^^38^^,^^39^, esta modalidad implica que personas con más experiencia “acompañen” a otras más jóvenes a domicilios o consultorios particulares en los que se realizan estas prácticas por fuera del sistema de salud en condiciones sanitarias deficientes y con personal poco capacitado, con los consiguientes riesgos para la salud que esto implica. Ello se condice con los datos relevados en nuestra investigación, en tanto 4 de cada 5 feminidades trans que se realizaron intervenciones no hormonales se aplicaron inyecciones de aceites y/o silicona líquida a temprana edad y en domicilios particulares.

En muchos casos, esas trayectorias de intervenciones corporales autogestionadas se constituyen como herramientas para la fluctuación, construcción y reconstrucción de la subjetividad. La tecnología cobra un sentido utilitario, pragmático y político, que acompaña un tránsito de enunciación subjetiva y la puesta en acto de modelos corporales divergentes que pueden incluir una apropiación desafiante y polémica^74^. Los datos relevados se condicen con un elevado porcentaje de efectos no deseados que muchas veces se conjuga con falta de información, con ausencia de monitoreo y de controles médicos propios de las modalidades de autoatención. Sin embargo, algunas prácticas implican efectos no deseados que requieren intervenciones sanitarias específicas, como es el caso de las inyecciones de aceites y/o silicona líquida. En ese sentido, los datos presentados permiten identificar algunos aspectos desatendidos en las políticas públicas en lo que refiere especialmente al acceso a cirugías y tratamiento de las secuelas del uso de aceites y/o silicona líquida. Consideramos que estos resultados permiten contribuir al desarrollo de intervenciones clínicas que den respuesta a las necesidades en salud de la población travesti y trans hasta el momento no reconocidas de manera programática.

Como resultado del trabajo surgen una serie de interrogantes a responder en futuros trabajos con el fin de comprender la complejidad del escenario local. Uno de ellos refiere a indagar qué dinámicas adquieren las redes y soportes comunitarios, institucionales y activistas que permiten sostener los cuidados en salud y mejorar la circulación de información al interior de esta población. Otra línea se orienta a identificar las distintas necesidades e itinerarios en salud de los diversos subgrupos, en especial de las personas que se identifican con otras identidades no cis. Finalmente, los emergentes de este trabajo dan cuenta de la importancia de las políticas de ampliación de derechos humanos en clave interseccional para promover una reducción de las inequidades en salud en las poblaciones históricamente vulneradas.
