# Supplementary material for: Tecnologías de modificación corporal y personas travestis y trans en Argentina: un estudio cuantitativo sobre desigualdades en el uso y acceso
Source: Salud Colect. 2025 Mar 21;21:e5206. [Article in Spanish] doi: 10.18294/sc.2025.5206 (PMC12303868; doi:10.18294/sc.2025.5206)
Supplement: Translation

# Translation: Body modification technologies and transvestite and trans people in Argentina: A quantitative study on inequalities in use and access

**Authors:** Anahí Farji Neer, María Alejandra Dellacasa, Sebastián Ezequiel Sustas, Melina Antoniucci, Cecilia Rustoyburu, Clara Inés Noceti, Natacha Mateo, Alejandra Rosario Roca

**Affiliations:** 1 PhD in Social Sciences. Assistant Researcher, Consejo Nacional de Investigaciones Científicas y Técnicas, based at Instituto de Estudios Sociales en Contextos de Desigualdades, Universidad Nacional de José C. Paz, Buenos Aires, Argentina.. anahi.farji@gmail.com; 2 PhD in Anthropology, Assistant Researcher, Consejo Nacional de Investigaciones Científicas y Técnicas, based at Instituto de Geografía, Historia y Ciencias Sociales, Universidad Nacional del Centro de la Provincia de Buenos Aires, Buenos Aires, Argentina. maledellacasa@yahoo.com.ar; 3 PhD in Social Sciences. Assistant Researcher, Consejo Nacional de Investigaciones Científicas y Técnicas, based at Instituto de Estudios para el Desarrollo Productivo y la Innovación, Universidad Nacional de José C. Paz, Buenos Aires, Argentina. s.sustas@gmail.com; 4 Degree in Sociology. Doctoral Fellow, Consejo Nacional de Investigaciones Científicas y Técnicas, based at Instituto de Investigaciones sobre Sociedades, Territorios y Culturas, Universidad Nacional de Mar del Plata, Mar del Plata, Argentina. melina.antoniucci@gmail.com; 5 PhD in Social Sciences. Associate Researcher, Consejo Nacional de Investigaciones Científicas y Técnicas, based at Instituto de Investigaciones sobre Sociedades, Territorios y Culturas, Universidad Nacional de Mar del Plata, Mar del Plata, Argentina. ceciliarustoyburu@gmail.com; 6 Physician, Specialist in General and Family Medicine. Centro de Salud y Acción Comunitaria No. 28, Hospital General de Agudos Donación Francisco Santojanni, Buenos Aires, Argentina. clara.noceti@gmail.com; 7 PhD in Social Communication. Postdoctoral fellow, Consejo Nacional de Investigaciones Científicas y Técnicas, based at Instituto de Investigaciones sobre Sociedades, Territorios y Culturas, Universidad Nacional de Mar del Plata, Mar del Plata, Argentina. mateonatacha@gmail.com; 8 PhD in Anthropology. Research Professor, Institute of Studies for Productive Development and Innovation, National University of José C. Paz, Buenos Aires, Argentina. roca.ale@gmail.com

**Keywords:** Transgender Persons, Gender Identity, Hormones, Surgery, Silicone Oils, Argentina

## Abstract

Collective health has provided valuable analytical perspectives for understanding the social determinants of health and how dynamics of inequality, inequity, and injustice contribute to suffering, disease, morbidity, and mortality. From this perspective, this study identifies and analyzes the ways in which transvestite and trans people in Argentina appropriate and use different hormones and body modification interventions, aiming to highlight the vulnerabilities associated with these practices, both those performed outside the healthcare system and those carried out with professional support in an institution. A quantitative methodological strategy was adopted, with a descriptive, observational, and cross-sectional design. In 2023, a nationwide survey was conducted using a questionnaire targeting transvestite and trans people aged 16 and older (n=1,196). The analyzed data reveal vulnerability processes that do not extend uniformly across the entire population and are linked to health inequalities.

## INTRODUCTION

Trans experiences, shaped at the intersection of modern medicalizing and pathologizing discourses and those of the subjects themselves, are deeply connected to the development of biomedical body modification technologies (such as surgeries and implants), the consolidation of endocrinology as a medical specialty, and the expansion of the pharmaceutical industry.^1^ From the mid-20th century to the early 21st century, particularly in the European and U.S. contexts, the biomedical field’s assignment of the diagnosis of “transsexualism” or “gender identity disorder” required individuals to express an unequivocal desire and willingness to modify their bodies in order to “align” them with the desired gender. These processes legitimized a narrative that linked trans lives with psychological and physical suffering. However, other studies have documented narratives in which experimentation, pleasure, or aesthetics prevail^2^^,^^3^^,^^4^^,^^5^ and have highlighted how these requirements forced individuals to replicate a pre-established script to obtain authorization for access to surgeries and hormone therapies.^6^^,^^7^^,^^8^ Other approaches have also shown that, for trans individuals who seek these procedures, surgeries and hormone therapies constitute the most suitable means to prevent self-harm and suffering, ultimately improving their quality of life.^9^^,^^10^

Criticism of the pathologization of trans experiences is a central axis of transgender studies.^11^ This field originated in the United States in the 1990s, with foundational texts offering profound critiques of the medical field’s imposed protocols for accessing body modification procedures.^8^^,^^12^ Within this field, the concept of *cissexual privilege*^13^ was developed to account for a specific regime that oppresses trans people. The historical silencing of trans voices regarding their own bodies and lives, a constitutive feature of cissexism, occupies a central place in the debates within this area of study.^14^

Since the 2000s, activist movements have promoted the depathologization of trans experiences, advocating for the recognition that they should no longer be understood as disorders. Through various initiatives and public demonstrations worldwide, activists have demanded that access to body modification therapies and interventions no longer require lengthy and humiliating diagnostic certification processes. Additionally, they have challenged the imposition of a single bodily model, calling for individuals to freely decide which interventions to undergo, if any, and at what point in their lives.^15^^,^^16^^,^^17^^,^^18^ This stance has sparked debate between those who argue that access to these practices should be framed as a health issue and those who interpret them as purely aesthetic.^19^ Other perspectives have focused on the autonomy and agency of trans individuals, viewing surgeries as interventions embedded in a process of negotiation and democratization of access to technology, thus transcending the normalization of bodies.^20^ Further research has also explored the subjective dimensions of surgical interventions, emphasizing the meanings and sense of “rebirth” these procedures entail for trans people,^21^ as well as the psychological and contextual factors that influence decision-making regarding body modifications within this population.^22^

### Background on body modification technologies among travesti and trans populations in Argentina and Latin America

Ethnographic research has been instrumental in documenting the strategies for constructing “travesti bodies” in different regions of Argentina, Brazil, and Colombia.^23^^,^^24^^,^^25^^,^^26^^,^^27^^,^^28^ Within this line of inquiry, studies have highlighted the persistence of practices that, due to their unintended effects, can be hazardous. Although the use of hormones without medical support poses significant health risks,^29^^,^^30^^,^^31^^,^^32^^,^^33^ it remains a common practice among trans femininities. Similarly, the injection of liquid silicone and/or industrial oils to shape the body, performed outside the formal healthcare system, is also widespread. Numerous studies have explored the cultural meanings of these technologies, the reasons why individuals engage with them, and their negative health effects,^27^^,^^34^^,^^35^^,^^36^ revealing their entanglement with broader processes of social exclusion. In this regard, some quantitative surveys have found a high prevalence of these practices among travesti and trans feminine populations^37^ and have correlated them with sociodemographic, occupational, and educational data.^38^^,^^39^

In Argentina, while no federal-level quantitative studies with such large samples had previously been conducted, earlier research by travesti and trans organizations and provincial agencies had provided insights into the situation. A survey coordinated by Lohana Berkins in 2007^40^ reported that 86% of trans feminine respondents had injected liquid silicone, and 90% had undergone the procedure in a private residence. It also noted that 70% had initiated hormone therapy, yet only 20% had obtained the medication through the healthcare system. In 2017, a survey by the Public Defender’s Office of the Autonomous City of Buenos Aires^41^^)^ also identified the use of liquid silicone without medical support. However, it found that 25% of travestis and trans women who had undergone hormone treatments accessed hormones through the healthcare system, while 15% did so within the public health subsystem. The study also identified differences based on gender identity. Among trans masculinities, 61% accessed hormones through public hospitals, and only 4% self-administered them. Within this same population group, 88% reported modifying their bodies, with 97% undergoing hormone therapy. Additionally, 7% had undergone genital surgeries, and nearly half had undergone mastectomies.

In Argentina, the aforementioned debates led to the passage of Law 26.743 on Gender Identity in 2012. This law, drafted by local travesti and trans activists, establishes that no medical, judicial, or administrative authorization - nor prior surgical interventions - are required to request the legal change of name and sex on identification documents. Furthermore, the law mandates that therapies or interventions aimed at expressing gender identity must be covered by all three healthcare subsystems: public, private, and social security-based institutions. The Gender Identity Law, along with earlier initiatives such as the “Sexual Diversity-Friendly Clinics” consolidated in 2010^42^^,^^43^^,^^44^^,^^45^, sought to influence public policy in order to address the informal and often hazardous conditions in which these body modification processes take place.

### Health inequalities, risk, vulnerability, and self-care

In the field of collective health, health-illness-care processes are understood within the framework of hegemonic/subaltern relations, considering individuals as active agents in defining health-related practices and knowledge.^46^^,^^47^^,^^48^ Drawing from epistemologies of the Global South, collective health has provided rich analytical perspectives for understanding the social determinants of health in relation to economic and social inequalities. In this regard, the characterization of the notions of difference, distinction, inequality, inequity, and iniquity is essential.^49^ From this perspective, two key approaches can be articulated: on the one hand, the recognition of natural or genetic differences resulting from the interaction between social and biological dynamics; on the other, the acknowledgment that capitalist domination processes are at the root of material inequalities. Within a structural framework of domination, inequalities become inequities because they represent avoidable and unjust disparities. When these inequities are also morally unacceptable, they constitute iniquities. In relation specifically to health, this perspective posits that dynamics of inequality, inequity, and iniquity contribute to suffering, illness, morbidity, and mortality. This framework also incorporates the symbolic-cultural dimension, recognizing that individuals and groups affirm their identities by distinguishing themselves from others through specific tastes, rituals, behavioral patterns, and ways of life that impact their health.^50^^,^^51^

The factors determining health inequalities include access to material resources, lifestyle - understood as the intersection of living conditions and behavioral patterns - and the ability to access healthcare systems. From this perspective, it is crucial to consider the role of the state in either perpetuating or transforming social inequalities that affect health. Public policies help define the contours of citizenship, as they establish mechanisms of inclusion and exclusion within the political community, shaping the rights individuals can exercise, including the right to healthcare.^52^^,^^53^ Based on these considerations, this study understands gender, age, educational level, and whether an individual has legally changed their gender marker under Law 26.743 on Gender Identity as key dimensions shaping inequalities in the use of and access to body modification technologies, both within formal and informal contexts.

In examining and analyzing data that include body modification practices performed outside formal institutions, which may pose potential health risks, this study engages with critiques from the field of collective health regarding the notion of risk as conceptualized by traditional epidemiology. Specifically, it challenges the understanding of risk as an inherently biological and individual phenomenon that is objectively quantifiable, often overlooking the social conditions affecting individuals. Furthermore, it critiques the tendency of this epidemiological framework, under the guise of neutrality, to produce moralizing and stigmatizing narratives.^54^^,^^55^ As Castiel and Álvarez-Dardet^54^ argue, critical scrutiny is necessary when evaluating interventions aimed at mitigating behaviors deemed dangerous to individuals or their surroundings. While harm reduction interventions are necessary, they should avoid moralizing discourses of blame and be grounded in validated clinical guidelines and procedures.^54^

Unlike the risk-based approach, collective health integrates the perspective of vulnerability to examine the individual, social, and programmatic factors that contribute to health vulnerabilities and the possible strategies to address them.^54^^,^^56^^,^^57^ The individual dimension refers to how people build relationships - whether within communities, friendships, families, or professional networks - through which they co-construct and contest social meanings. The social dimension focuses on structural conditions, discourses, and values that shape individual vulnerabilities. Finally, the programmatic dimension considers the role of health policies and institutions, as well as those related to education, culture, and justice, in either reinforcing or mitigating individuals’ vulnerability conditions.

Collective health thus seeks to understand health-disease-care processes from an interdisciplinary perspective, as a totality that includes ways of life, practices, and meanings constructed by individuals whose health conditions are affected.^58^ From the perspective adopted in this study, we understand body modification practices as constitutive elements of the ways of life of travesti and trans populations. These practices are shaped by collectively constructed meanings about the body and identity, and they have various effects on the dynamics of health-disease-care.

In relation to practices and interventions that are at times carried out outside formal healthcare settings, it is also important to engage with what Eduardo Menéndez has described as processes of self-care:^59^ the articulation of different forms of knowledge and care carried out by individuals and social groups as active agents in the construction of their health. These practices should be taken into account by formal healthcare services in order to design more effective care strategies.^59^ This presents a challenge, especially considering that, due to the bias inherited from the hegemonic paradigm of the health sector, health demands that cannot be objectified within pathological frameworks often struggle to be recognized as legitimate health needs.^60^ Added to this bias are the cisnormative cultural logics that fail to acknowledge the bodily desires and life projects of travesti and trans people as legitimate. In the field of health - as in other professional fields - these logics are reproduced through the uncritical transmission of content during professional training.^61^

In this context, we find the notion of *accompaniment* more appropriate than that of *medical support*, as it recognizes individuals’ specific trajectories, their agency, and the legitimacy of the knowledge collectively produced by this population.

In light of these frameworks and debates, this article identifies and analyzes the ways in which travesti and trans people in Argentina appropriated and used various hormones and body modification interventions in 2023. The aim is to shed light on the vulnerabilities that may be involved in these practices, both those carried out outside the healthcare system and those that included professional accompaniment within institutions.

Moreover, this study considers a wide range of body modification procedures, acknowledging - as previous research has also indicated^4^^,^^62^^)^ - that while genital surgeries remain relevant, they have lost their centrality as gender markers and are now part of a broader set of interventions. In this sense, the voice, hair, chest and breasts, bones, skin, or face are all read as indicators of sexual difference that can be modified. We focus on the use of hormones, silicone implants, injections of industrial oils and/or liquid silicone, genital surgeries, botox injections, Adam’s apple reduction, speech therapy, among others. Our interest lies not only in highlighting the agency of travesti and trans individuals in appropriating and using these technologies, but also in critically examining how these practices are mediated by health inequalities and embedded in specific health-disease-care dynamics.

## MATERIALS AND METHODS

This research adopted a quantitative methodological strategy with a descriptive, observational, and cross-sectional design. A non-probabilistic, purposive sampling approach was used, based on partnerships with key informants and organizations of travesti and trans individuals from different regions of the country to construct the sample. This sampling strategy is often useful for describing phenomena within specific populations that are difficult to reach or engage.^63^^,^^64^ Quotas were established by gender identity and geographic region, based on secondary data from the National Registry of Persons (RENAPER)^65^ regarding legal gender recognition. Age was also included as a criterion in order to capture generational variation in the phenomena under study.

The sampling size strategy combined maximizing outreach through snowball sampling starting with territorial links to travesti and trans organizations, LGBT focused healthcare services, and institutions that serve as points of reference for the target population. A total of 1,196 individuals completed the questionnaire.

The data collection instrument included items related to police harassment, diagnosis and treatment of both non-communicable and communicable chronic diseases, hormone use, body modification interventions, modalities of use, quality of care, and access to the healthcare system among travesti and trans individuals residing in Argentina. A sociodemographic section was also included.

The development of the instrument involved several steps: reviewing and adapting items from instruments used in related studies;^66^^,^^67^^,^^68^ adapting previously validated tools for measuring access to and use of healthcare services;^69^^,^^70^^,^^71^ and conducting face and content validation through seven meetings of the research team held between July and September 2023 with members of civil society organizations of travesti and trans individuals, as well as two meetings with healthcare professionals experienced in working with this population across the different regions included in the study. A pilot test was subsequently conducted with travesti and trans individuals from participating organizations.

This process led to the final version of the questionnaire used in the fieldwork. These steps ensured the legitimacy of the instrument, the data collection process, and subsequent analysis. They also allowed for adjustments in phrasing, sequencing, and thematic organization; the inclusion of items relevant to the target population - as well as to the healthcare personnel who typically provide their care - and the conceptual and operational alignment of the questionnaire.^72^

For the questionnaire’s implementation in the field, travesti and trans organizations and sex worker groups from different regions of the country were invited to participate, in order to ensure their involvement throughout the research process.

The questionnaire was programmed using the online platform Survey Monkey, which enabled the creation of filters and skip logic between questions. It also ensured the anonymity and confidentiality of respondents’ data. The questionnaire consisted of 87 questions, including an informed consent item, and was composed almost entirely of closed-ended questions, including single-choice, multiple-choice, and numerical response formats.

Field access was ensured through the prior process of participation and collaboration with the aforementioned organizations. Programming the instrument in online software enabled the questionnaire to be administered individually and face-to-face using personal mobile devices. The organization of fieldwork involved different roles: members of the research team were responsible for liaising with organizations and monitoring fieldwork. Depending on the number of assigned cases, field coordinators were designated, along with individuals responsible for administering the questionnaires within each organization. Training sessions were conducted to support the implementation of the instrument. Each interviewer was provided with mobile data to access the internet on their phone, which was used to administer the survey.

The data obtained through the survey were analyzed using univariate and bivariate analyses. For the bivariate analysis, we considered the following independent variables: gender identity, grouped age, highest educational attainment, and whether or not the respondent had legally changed their registered name and gender in their National Identity Document (DNI). Regarding gender identity, the questionnaire asked about self-perceived identity, offering the following response options: trans woman, trans femininities, travesti, trans man, trans masculinities, transgender, transsexual, non-binary, gender fluid, and other identity. In order to generate a consistent variable that would reflect both identity and bodily modification trajectories, we regrouped responses into three categories: trans femininities, trans masculinities, and other non-cisgender identities. This methodological decision also enables comparisons with previous studies .^41^^,^^66^

The “trans femininities” category included trans women, trans femininities, travestis, and individuals assigned to the transsexual category; “trans masculinities” included trans men, trans masculinities, and individuals assigned to the transsexual category; and “other non-cisgender identities” included transgender, non-binary, gender fluid, and other identities. Highest educational attainment was included as a proximal variable for social stratification and was recategorized into three groups: those who had completed up to primary school, those who had attended or completed secondary education, and those who had accessed tertiary or higher education. In addition, legal gender recognition was considered a proximal variable for programmatic and institutional support.

The dependent variables used were: having ever undergone hormone replacement therapy (HRT); presence of medical accompaniment during the first HRT; who provided guidance in the absence of medical accompaniment; the presence and types of adverse effects resulting from the first HRT; and whether the respondent had been informed of potential side effects. Additional dependent variables included: having undergone any type of body modification procedure; the types of procedures undertaken; having received injections of liquid silicone and/or industrial oils; whether these caused any adverse effects and what kind; whether there was a need or desire to have these substances removed; whether there was an intention to undergo other body modification procedures and which ones; and the reasons for not having done so yet.

The data were processed and analyzed using SPSS software. Contingency tables were used to compare the categories of independent variables using Pearson’s chi-square test (χ²). This non-parametric test is appropriate for the type of nominal variables used in the study, as it allows for the assessment of whether observed distributions differ from expected ones in the cross-tabulations of dependent and independent variables.^63^ Statistical significance was set at p <0.05.

After presenting the purpose of the research, respondents were informed about the anonymous and confidential treatment of the data collected. An informed consent question was included at the beginning of the questionnaire, and a supplementary information sheet was also made available. Although no personal data were collected, safeguards were implemented to avoid duplicate responses by cross-referencing the completion date, age, gender, and place of birth. The research team did not have access to any identifying information of participants. The project was approved on August 28, 2023, by the Bioethics Committee of the National University of Mar del Plata, Buenos Aires, Argentina.

## RESULTS

### Sample description

The final sample consisted of 1,196 individuals (Table 1). Approximately one-third resided in the Buenos Aires Metropolitan Area, 39.1% in the Central region, and 8.9% in the Northwestern region. The Northeastern, Cuyo, and Patagonian regions each accounted for 6% of respondents. A total of 59.3% identified as trans femininities, 25.4% as trans masculinities, and 15.3% as other non-cisgender identities.

**Table 1:** Sample characteristics by gender identity, grouped age, region of residence, highest educational level attained, and legal gender marker change. Argentina, 2023.

Variables	n	%
**Gender identity**		
Trans femininities	709	59.3
Trans masculinities	304	25.4
Other non-cisgender identities	183	15.3
**Grouped age**		
16-24	292	24.4
25-34	435	36.4
35-44	284	23.7
45-54	144	12.0
55 and over	41	3.4
**Region**		
Center	468	39.1
AMBA	410	34.3
NOA	107	8.9
Patagonia	73	6.1
NEA	72	6.0
Cuyo	66	5.5
**Highest educational level**		
Primary	230	19.2
Secondary	664	55.5
Higher	302	25.3
**Legal gender marker change**		
Yes	837	70.0
No	359	30.0

Source: Own elaboration based on data collected in the original research. AMBA = Buenos Aires Metropolitan Area; NOA = Argentine Northwest; NEA = Argentine Northeast.

The age distribution is likely related to specific features of the health, illness, and care trajectories experienced by this population,^41^^,^^67^^,^^68^ and reflects a relatively young sample: 60.8% were under the age of 35, 23.7% were between 35 and 44 years old, 12.0% between 45 and 54, and only 3.4% were over 55.

In terms of educational attainment, 19.2% had completed primary education, 55.5% had attended or completed secondary education, and 25.3% had accessed tertiary or higher education (regardless of whether they completed the level). Finally, more than two-thirds of respondents had completed the legal process of changing their name and gender marker on their national identity document.

### Hormone therapy processes

To explore experiences of hormone use and access to hormone therapy processes, we asked respondents whether they had ever undergone hormone replacement therapy (HRT) related to their gender identity and expression (Table 2). Over half of the respondents answered affirmatively (n=670, 56.0%). When considering gender identity as an independent variable, this proportion rises to 67.4% among trans masculinities and 59.5% among trans femininities, while it drops to 23.5% among those with other non-cisgender identities (*p*=0.001).

**Table 2:** Lifetime use of hormone replacement therapy by gender identity, grouped age, highest educational level attained, and legal gender marker change. Argentina, 2023.

Variables	Total (n=1,196, 100.0%)	Used HRT (n=670, 56.0%)		Did not use HRT (n=526, 44.0%)		p-value
	n	n	%	n	%	
**Gender identity**						
Trans masculinities	304	205	67.4	99	32.6	0.001
Trans femininities	709	422	59.5	287	40.5	
Other non-cisgender identities	183	43	23.5	140	76.5	
**Grouped age**						
16-24	292	166	56.8	126	43.2	0.431
25-34	435	255	58.6	180	41.4	
35-44	284	154	54.2	130	45.8	
45-54	144	76	52.8	68	47.2	
55 and over	41	19	46.3	22	53.7	
**Highest educational level**						
Primary	230	95	41.3	135	58.7	0.001
Secondary	664	378	56.9	286	43.1	
Higher	302	197	65.2	105	34.8	
**Legal gender marker change**						
No	359	116	32.3	243	67.7	0.001
Yes	837	554	66.2	283	33.8	

Source: Own elaboration based on data collected in the original research. HRT = Hormone replacement therapy. Note: p-value from χ^2^ test.

Regarding educational attainment, we observed that the proportion of individuals who had undergone hormone replacement therapy increased with higher levels of education: 41.3% among those with primary education, 56.9% among those with secondary education, and 65.2% among those who accessed higher education (*p*=0.001).

Finally, 66.2% of respondents who had legally changed their gender marker and name on their identity documents reported having undergone hormone therapy. Among those who had not carried out the legal change, only 32.3% had received such therapy (*p*=0.001).

We asked whether this first hormone therapy experience was carried out with the accompaniment of a healthcare team (Table 3). Among those who had undergone hormone therapy, 60.0% (n=402) reported that their first experience was medically supervised. When analyzing the relationship between age groups and medical accompaniment, we observed that the proportion of individuals who received medical accompaniment decreases as age increases (*p*=0.001).

**Table 3:** First hormone replacement therapy with medical accompaniment at a healthcare institution, by gender identity, grouped age, highest educational level attained, and legal gender marker change. Argentina, 2023.

Variables	Total (n=670, 100.0%)	With medical accompaniment (n=402, 60.0%)		Without medical accompaniment (n=268, 40.0%)		p-value
	n	n	%	n	%	
**Gender identity**						
Trans masculinities	205	187	91.2	18	8.8	0.001
Trans femininities	422	182	43.1	240	56.9	
Other non-cisgender identities	43	33	76.7	10	23.3	
**Grouped age**						
16-24	166	137	82.5	29	17.5	0.001
25-34	255	168	65.9	87	34.1	
35-44	154	67	43.5	87	56.5	
45-54	76	26	34.2	50	65.8	
55 and over	19	4	21.1	15	78.9	
**Highest educational level**						
Primary	95	33	34.7	62	65.3	0.001
Secondary	378	221	58.5	157	41.5	
Higher	197	148	75.1	49	24.9	
**Legal gender marker change**						
No	116	58	50.0	58	50.0	0.016
Yes	554	344	62.1	210	37.9	

Source: Own elaboration based on data collected in the original research. Note: p-value from χ^2^ test.

Significant differences were also observed when considering gender identity: 91.2% of trans masculinities received professional medical accompaniment during their first hormone therapy experience, compared to only 43.1% of trans femininities (*p*=0.001).

Analyzing educational attainment, we found that the proportion of individuals who received medically supervised hormone therapy in institutional healthcare settings was higher among those with higher levels of education (*p*=0.001). Finally, among those who had carried out the legal gender marker change, 62.1% reported having medical accompaniment during their first hormone therapy experience, compared to 50.0% of those who had medical accompaniment but had not carried out the legal change (*p*=0.016).

Among those who had undergone hormone therapy at some point, 40.0% (n=268) did so without medical accompaniment. When asked who had provided guidance or advice when starting hormone therapy, the vast majority responded that it came from friends (77.6%). The remaining responses were distributed in proportions under 10%, including acquaintances, members of organizations, partners, “godmothers,” family members, pharmacists, or information found online or on social media.

Returning to the group of individuals who had ever undergone HRT, 26.0% (n=174) reported experiencing unwanted side effects during their first hormone replacement therapy (Table 4). The proportion of individuals reporting such effects varied across the independent variables considered: the proportion was higher among older individuals (although not statistically significant), and particularly among those categorized under other non-cisgender identities (37.2%). A higher proportion of unwanted effects was also reported among individuals with lower educational attainment (34.7%).

**Table 4:** Undesired effects of first hormone replacement therapy by gender identity, grouped age, highest educational level attained, and legal gender marker change. Argentina, 2023.

Variables	Total (n=670, 100.0%)	Undesired effects (n=174, 26.0%)		No undesirable effects (n=447, 66.7%)		Do not remember (n=49, 7.3%)		p-value
	n	n	%	n	%	n	%	
**Gender identity**								
Trans femininities	422	127	30.1	265	62.8	30	7.1	0.001
Trans masculinities	205	31	15.1	159	77.6	15	7.3	
Other non-cisgender identities	43	16	37.2	23	53.5	4	9.3	
**Grouped age**								
16-24	166	36	21.7	118	71.1	12	7.2	0.701
25-34	255	66	25.9	173	67.8	16	6.3	
35-44	154	41	26.6	99	64.3	14	9.1	
45-54	76	25	32.9	46	60.5	5	6.6	
55 and over	19	6	31.6	11	57.9	2	10.5	
**Highest educational level**								
Primary	95	33	34.7	48	50.5	14	14.7	0.001
Secondary	378	91	24.1	269	71.2	18	4.8	
Higher	197	50	25.4	130	66.0	17	8.6	
**Legal gender marker change**								
Yes	554	137	24.7	378	68.2	39	7.0	0.190
No	116	37	31.9	69	59.5	10	8.6	

Source: Own elaboration based on data collected in the original research. Note: p-value from χ^2^ test.

When asked about the specific unwanted effects that impacted their health, 38.5% referred to mood changes (including symptoms such as anger, anxiety, depression, or insomnia). These effects were more frequently reported by trans femininities and other non-cisgender identities. Other unwanted effects included liver and/or stomach issues (19.0%), which were more commonly reported among trans femininities. These were followed by skin problems (12.6%), mainly reported by trans masculinities, and changes in body weight (12.1%). Additional reported effects included sexual dysfunctions, unspecified pain, changes in hair and body hair distribution, circulatory problems, hormonal imbalances, and changes in body composition.

Among those who experienced unwanted effects during their first hormone therapy process, we asked whether they had been informed about the potential adverse effects of hormone treatments (Table 5). A total of 43.7% (n=76) responded affirmatively. This proportion was higher among trans masculinities (64.5%), individuals identifying with another non-cisgender identity (62.5%), and those aged 16 to 24 years (61.1%). As age increased - and with it the time elapsed since the first hormone therapy experience - the proportion of individuals who had received information about potential adverse effects decreased. A lower proportion of respondents who had been informed was found among trans femininities (36.2%) and individuals with lower levels of education (33.3%).

**Table 5:** Information received about undesired effects of first hormone replacement therapy by gender identity, grouped age, highest educational level attained, and legal gender marker change. Argentina, 2023.

Variables	Total (n=174, 100.0%)	Received information (n=76, 43.7%)		Did not receive information (n=88, 50.6%)		Do not remember (n=10, 5.7%)		p-value
	n	n	%	n	%	n	%	
**Gender identity**								
Trans femininities	127	46	36.2	75	59.1	6	4.7	0.007
Trans masculinities	31	20	64.5	9	29.0	2	6.5	
Other non-cisgender identities	16	10	62.5	4	25.0	2	12.5	
**Grouped age**								
16-24	36	22	61.1	10	27.8	4	11.1	0.025
25-34	66	33	50.0	30	45.5	3	4.5	
35-44	41	13	31.7	26	63.4	2	4.9	
45-54	25	7	28.0	17	68.0	1	4.0	
55 and over	6	1	16.7	5	83.3	0	0.0	
**Highest educational level**								
Primary	33	11	33.3	20	60.6	2	6.1	0.673
Secondary	91	44	48.4	42	46.2	5	5.5	
Higher	50	21	42.0	26	52.0	3	6.0	
**Legal gender marker change**								
No	37	16	43.2	20	54.1	1	2.7	0.646
Yes	137	60	43.8	68	49.6	9	6.6	

Source: Own elaboration based on data collected in the original research. Note: p-value from χ^2^ test.

### Other interventions related to gender identity and expression

Among the total number of people who completed the questionnaire, 46.1% (n=551) reported having undergone another type of bodily intervention related to their gender identity and expression (Table 6). More than half of those who identified as trans femininities had undergone this type of intervention (56.3%), compared to 38.2% of those who identified as trans masculinities and 19.7% of those with other non-cisgender identities (*p*=0.001). Over 60% of respondents aged between 35 and 54 answered affirmatively to this question, as did 51.2% of those over 55. In contrast, only 19.2% of individuals aged 16 to 24 had undergone any such interventions (*p*=0.001). Among those who had carried out the legal gender marker change, 55.6% had undergone at least one intervention, compared to 24.0% of those who had not (*p*=0.001). No significant differences were found in this dimension when analyzed by highest educational attainment.

**Table 6:** Other body modification interventions, by gender identity, grouped age, highest educational level attained, and legal gender marker change. Argentina, 2023.

Variables	Total (n=1,196, 100.0%)	Performed OBMI (n=551, 46.1%)		Did not perform OBMI (n=645, 53.9%)		p-value
		n	n	%	n		%
**Gender identity**						
Trans masculinities	304	116	38.2	188	61.8	0.001
Trans femininities	709	399	56.3	310	43.7	
Other non-cis identities	183	36	19.7	147	80.3	
**Grouped age**						
16-24	292	56	19.2	236	80.8	0.001
25-34	435	215	49.4	220	50.6	
35-44	284	172	60.6	112	39.4	
45-54	144	87	60.4	57	39.6	
55 and over	41	21	51.2	20	48.8	
**Highest educational level**						
Primary	230	103	44.8	127	55.2	0.341
Secondary	664	318	47.9	346	52.1	
Higher	302	130	43.0	172	57.0	
**Legal gender marker change**						
No	359	86	24.0	273	76.0	0.001
Yes	837	465	55.6	372	44.4	

Source: Own elaboration based on data collected in the original research. OBMI= Other body modification interventions. Note: p-value from χ^2^ test.

Among those who had undergone some type of intervention, the most commonly reported procedures were: breast implants (54.8%), mastectomy with pectoral reconstruction (22.7%), facial modification surgery (21.8%), gluteal implants (18.0%), and botox injections (14.0%). In contrast, vaginoplasty was mentioned by only 4.7% and phalloplasty by 0.5%. Approximately 72.9% of respondents who identified as trans femininities had received breast implants, while more than 92.2% of those identifying as trans masculinities had undergone mastectomy with pectoral reconstruction.

Participants were also asked whether they intended to undergo any bodily intervention in the future (Table 7). A total of 61.4% (n=734) of the sample responded affirmatively. This proportion was higher among trans femininities (64.7%) and slightly higher among trans masculinities (61.8%), while it was lower among people with other non-cisgender identities (47.5%) (*p*=0.001). When analyzed by age group, a statistically significant association was also found (*p*=0.005): the intention to undergo bodily interventions was greater among younger respondents (16-24 years) and lower among those over 45. Incorporating educational attainment revealed a clear pattern: the intention to undergo some type of intervention increases with higher levels of education (*p*=0.001). When analyzed by legal gender marker change status, a higher intention was observed among those who had completed the process (64.0%), compared to those who had not (55.2%) (*p*=0.004).

**Table 7:** Intention to undergo other body modification interventions, by gender identity, grouped age, highest educational level attained, and legal gender marker change. Argentina, 2023.

Variables	Total (n=1.196, 100.0%)	Intends to perform OBMI (n=734, 61.4%)		Does not intend to perform OBMI (n=462, 38.6%)		p-value
	n	n	%	n	%	
**Gender identity**						
Trans femininities	709	459	64.7	250	35.3	0.001
Trans masculinities	304	188	61.8	116	38.2	
Other non-cisgender identities	183	87	47.5	96	52.5	
**Grouped age**						
16-24	292	200	68.5	92	31.5	0.005
25-34	435	272	62.5	163	37.5	
35-44	284	165	58.1	119	41.9	
45-54	144	73	50.7	71	49.3	
55 and over	41	24	58.5	17	41.5	
**Highest educational level**						
Primary	230	117	50.9	113	49.1	0.001
Secondary	664	420	63.3	244	36.7	
Higher	302	197	65.2	105	34.8	
**Legal gender marker change**						
Yer	837	536	64.0	301	36.0	0.004
No	359	198	55.2	161	44.8	

Source: Own elaboration based on data collected in the original research. OBMI = Other body modification interventions. Note: *p*-value from χ^2^ test.

The most frequently mentioned procedures that participants expressed a desire to undergo were: breast implants (35.1%), facial modification surgery (32.8%), mastectomy with pectoral reconstruction (22.5%), vaginoplasty (18.4%), gluteal implants (14.4%), Adam’s apple reduction or chondrolaryngoplasty (12.9%), and botox injections (11.2%). Lastly, phalloplasty was mentioned by 9.8% - with a predominance among trans masculinities and a smaller percentage among other non-cisgender identities - and speech therapy by 9.9%, with a similar distribution across the different gender identities used as independent variables. Regarding gender identity, the selection of procedures among trans masculinities and trans femininities appeared to be shaped by binary representations of what constitutes a masculine or feminine body. Among people with other non-cisgender identities, there was interest in accessing chest surgeries (mastectomy or implants) and facial modification procedures.

Among those who expressed an intention to undergo an intervention in the future related to the construction of their body and gender expression, we explored the reasons why they had not yet done so. These reasons reflect potential access barriers as well as cases of personal postponement by choice. The main reasons cited by participants included: financial difficulties in accessing the procedures (39.5%), long waiting times (16.5%), perceived lack of trustworthy professionals (10.6%), and lack of training and/or specialists (5.6%). Among the personal obstacles, fear and/or apprehension toward the intervention (13.5%) and lack of knowledge about the institutional mechanisms for accessing the procedures (12.3%) were noted. Other challenges referred to negative personal or second-hand experiences with the healthcare system, such as knowing others who did not achieve the expected results following the intervention (4.1%) and previous negative experiences in healthcare institutions (3.4%).

### Injections with oils and/or liquid silicone

In addition to surgical interventions, we explored the use of liquid silicone injections or other types of oil-based injections for body modification (Table 8). Among those who had undergone some form of intervention, more than half reported having received such injections (50.6%, n=279). Among trans femininities, 67.4% reported having used oils and/or liquid silicone, while this figure dropped to 27.8% among individuals with other non-cisgender identities. None of the trans masculinities reported having used such injections (*p*=0.001). When considering age groups, the data show that individuals aged 35 and older were more likely to report having used oil and/or silicone injections, with this figure reaching 81.0% among those aged 55 and over (*p*=0.001). Regarding the highest level of education attained, the use of oil and/or silicone injections was more prevalent among individuals with lower levels of education (*p*=0.001). Lastly, among those who had completed the legal gender marker change on their identity documents, 48.6% reported having used oil and/or silicone injections, compared to 61.6% of those who had not made the legal change (*p*=0.026).

**Table 8:** Injection or use of oils and/or liquid silicone, by gender identity, grouped age, highest educational level attained, and legal gender marker change. Argentina, 2023.

Variables	Total (n=551, 100.0%)	Performed IOLS (n=279, 50.6%)		Did not perform IOLS (n=272, 49.4%)		p-value
	n	n	%	n	%	
**Gender identity**						
Trans femininities	399	269	67.4	130	32.6	0.001
Trans masculinities	116	0	0.0	116	100.0	
Other non-cisgender identities	36	10	27.8	26	72.2	
**Grouped age**						
16-24	56	13	23.2	43	76.8	0.001
25-34	215	82	38.1	133	61.9	
35-44	172	110	64.0	62	36.0	
45-54	87	57	65.5	30	34.5	
55 and over	21	17	81.0	4	19.0	
**Highest educational level**						
Primary	103	86	83.5	17	16.5	0.001
Secondary	318	164	51.6	154	48.4	
Higher	130	29	22.3	101	77.7	
**Legal gender marker change**						
Yes	465	226	48.6	239	51.4	0.026
No	86	53	61.6	33	38.4	

Source: Own elaboration based on data collected in the original research. IOLS = Injection or use of oils and/or liquid silicone. Note: *p*-value from χ^2^ test.

Those who reported having used silicone and/or oil injections were asked about the age at which the procedure took place. All respondents indicated they underwent the intervention before the age of 24. Regarding the setting in which the injections were administered, 85.3% reported having received them in a private residence. This context often involves the use of substances not suitable for human use, the absence of adequately trained personnel, and a lack of proper hygiene and aseptic conditions, all of which pose significant health risks.

Among those who had at some point injected oils and/or liquid silicone, 32.3% (n=90) reported experiencing some adverse effects after the procedure. This percentage increased with age, reaching 58.8% among people aged 55 and over, and 43.9% among those aged 45 to 54. While none of the individuals under 24 years of age reported adverse effects, 22.0% of those aged 25 to 34 and 33.6% of those aged 35 to 44 did report such outcomes (*p*=0.001). No statistically significant differences were found according to gender identity, highest educational level attained, or whether or not a legal gender marker change had been made.

The survey included an open-ended question about the specific adverse effects experienced. Ninety individuals responded, and nearly one-third described more than one condition, leading to the identification of 122 effects, which were then grouped into seven categories reflecting similar somatic processes. The most commonly reported issues included pain, inflammation, discomfort, and/or swelling (54.4%), typically in the legs; substance migration and resulting deformities (34.4%); bluish skin discoloration (14.4%); granulomas (10.0%); fever and/or infections (10.0%); and dizziness, nausea, and/or vomiting (4.4%). Less frequently mentioned effects -such as respiratory difficulties, uneven hips, varicose veins, and intestinal issues- were grouped under “other.”

Age was a relevant factor in the experience of adverse effects. Pain, inflammation, discomfort, and/or swelling were reported most frequently by individuals aged 55 and older, although roughly half of respondents in other age groups also mentioned these symptoms. Substance migration and deformities were most commonly reported by individuals aged 35 to 54.

Among those who had undergone these procedures, 75.6% had not received any subsequent medical follow-up. When analyzing by educational level, this proportion was highest among those with only primary education (79.1%) and decreased significantly among individuals with higher education (51.7%) (*p*=0.007). No statistically significant differences were found in relation to the other cross-tabulated variables. Finally, 40.1% of those who had used oils and/or liquid silicone reported needing or intending to undergo surgical removal of these substances.

## DISCUSSION AND CONCLUSIONS

From a perspective rooted in the field of collective health and informed by approaches from trans studies, we understand hormonal therapies and other body modification practices carried out by travesti and trans populations as interventions aimed at supporting their identity processes. From this standpoint, we now summarize the key aspects of the analysis presented, with a focus on the dynamics of inequality, risk/vulnerability, and self-care that can be identified.

As noted at the outset, we understand gender, age, educational level, and whether or not a person has undertaken legal gender marker change as dimensions that structure inequalities in the use of and access to body modification technologies. From the perspective of collective health, these inequalities constitute health inequities, as they imply differential exposure to suffering, illness, and phenomena of morbidity and mortality. The data on the travesti and trans population gathered in this study allows us to examine the ways in which differences in gender, age, educational level, and legal gender marker change shape different uses of body modification technologies. These uses are related to subjective and collective desires and expectations regarding bodily appearance and gender expression, but also to the availability of such technologies within the healthcare system and the ability of individuals to access them. In this sense, people with higher educational attainment had greater access to hormone therapies. This reflects the existence of subgroups within the travesti and trans population in terms of the support available for hormonal body modification processes, possibly linked to the unequal distribution of material resources within this population.

The data also reveal differing modalities of access to healthcare services. For example, the relationships identified between legal gender marker change and access to health services provide insight into the connections between individuals and institutions. People who had completed the legal gender marker change reported greater use of and access to bodily interventions, along with more follow-up and monitoring. Conversely, those who had not done so more frequently underwent interventions outside the formal healthcare system and had fewer health checkups. These findings make visible the rights that remain unfulfilled for travesti and trans people who have not completed legal gender marker change.

In Argentina, since the enactment of Law 26.743, public policy has shifted from a focus on “pathologization” and “compensatory” strategies toward a framework that positions travesti and trans individuals as “active subjects of rights.” We argue that the legal recognition of a person’s self-perceived gender identity is a fundamental right that provides institutional support and fosters full citizenship, as it enables the exercise of other rights (healthcare, education, formal employment, border mobility, among others). The data emerging from this study allow us to reflect on the relationship between the legal recognition of travesti and trans identities - enshrined in the 2012 Gender Identity Law - and the opening of channels for accessing the formal healthcare system. In other words, they allow us to trace a process whereby the enactment of legislation contributes to the transformation of social health inequalities.

The data analyzed also point to processes of vulnerability that do not affect the entire population uniformly. We found that trans femininities, older individuals, those with lower levels of education, and those who had not undergone legal gender marker change were the groups with the least access to medical accompaniment and the highest number of practices associated with potential adverse effects.

From the perspective adopted in this study, and based on our independent variables, we identified dimensions related to processes of vulnerability that influence exposure to body modification practices involving potential health risks or harm. These observed inequalities cannot be fully understood without referring to the structural inequities that produce them. Individual trajectories are embedded within a context marked by accumulated violations over time, which become embodied in the unequal access to the right to comprehensive health and in the various modes of access to and use of body modification technologies. For instance, we observed greater use of oil and/or silicone injections among older individuals. From a generational perspective, this suggests health-illness-care processes shaped by prolonged exposure to risky practices, which may be linked to an accumulation of vulnerabilities and violations.

Although existing studies at the national level^66^^,^^67^^,^^68^ differ in design and sample size, they allow us to identify some continuities in practices such as hormone use without medical accompaniment and the injection of oils and/or liquid silicone among trans femininities, particularly among those facing heightened socioeconomic and institutional vulnerability. Conversely, individuals with greater institutional support - whether through completing legal gender marker change or achieving higher educational levels - exhibit profiles marked by greater engagement with the formal healthcare system. This is also reflected in the reported health consequences: the most vulnerable groups are those who most frequently report adverse effects and/or a need for care due to the sequelae. Regarding hormone replacement therapy, the most vulnerable groups and trans femininities reported receiving less information about its potential side effects, revealing deficits in the quality of care. A generational reading further suggests a gap that may reflect changes in public policy and their impact on the organization of care services: younger generations report receiving more information from healthcare providers about potential negative effects of these therapies.

In relation to self-care modalities, we argue that as long as body modification processes among travesti and trans populations are not recognized as legitimate health needs, significant barriers remain to accessing such procedures in a supported, institutional framework that can provide care and protection. In this sense, although the enactment of the Gender Identity Law in Argentina marked a major advance in terms of rights and healthcare, the lack of healthcare spaces staffed with professionals trained in travesti and trans health throughout the country, as well as the limited availability of hormonal drugs and/or prostheses, significantly hinders the possibility of guaranteeing access in accordance with what the law mandates. According to our analysis, the main reasons why respondents had not yet carried out the body modification procedures they wished to undergo within the health system included: financial difficulties, long wait times, and a lack of trustworthy professionals. In contrast, over time - and particularly among older trans femininities, those with lower educational attainment, and those who had not completed legal gender marker change - a repertoire of practices has emerged outside of the formal healthcare system.

Among travesti and trans people, information frequently circulates through informal channels - both via social networks and word of mouth - regarding which hormones to take, in what doses and how often, to achieve certain bodily changes. This has led to widespread self-medication. Our data reflects this: a significant proportion of those who had undergone hormone replacement therapy did so without medical accompaniment. When asked who had guided or advised them at the time of starting HRT, the vast majority said it had been friends. Similarly, information circulates within this population about the use of various injectable substances - such as oils and liquid silicone - that are not fit for human use, and the practice has become widespread. As mentioned earlier, and as noted in other studies,^37^^,^^38^^,^^39^ this practice often involves more experienced individuals “accompanying” younger people to private homes or informal clinics where these procedures are carried out outside the health system, under poor sanitary conditions and by inadequately trained personnel, resulting in significant health risks. This is consistent with the data gathered in our study, which show that 4 out of 5 trans femininities who had undergone non-hormonal procedures had received injections of oils and/or liquid silicone at an early age and in private homes.

In many cases, these trajectories of self-managed bodily interventions serve as tools for the fluctuation, construction, and reconstruction of subjectivity. Technology assumes a utilitarian, pragmatic, and political meaning, accompanying a process of subjective enunciation and the enactment of divergent corporeal models that may involve a defiant and contentious appropriation.^74^ The data collected are consistent with a high percentage of adverse effects, often combined with a lack of information, absence of monitoring, and a lack of medical accompaniment-features characteristic of self-care modalities. However, some practices result in adverse effects that require specific health interventions, such as the injection of oils and/or liquid silicone. In this regard, the data presented allow us to identify certain areas neglected by public policies, particularly in terms of access to surgery and treatment for the consequences of oil and/or liquid silicone use. We believe that these findings contribute to the development of clinical interventions that address health needs of the travesti and trans population that have thus far not been recognized programmatically.

This study also raises a series of questions to be addressed in future research aimed at better understanding the complexity of the local context. One such question involves exploring the dynamics of community, institutional, and activist networks and support systems that help sustain healthcare practices and improve the circulation of information within this population. Another line of inquiry focuses on identifying the different health needs and trajectories among various subgroups, especially among individuals who identify with non-cisgender identities. Finally, the findings of this study underscore the importance of human rights-based policies, framed through an intersectional lens, to promote the reduction of health inequities in historically marginalized populations.
